# Optimisation and Validation of a Microscale FRAP Assay with Matrix-Matched (Interaction-Corrected) Blanking for Accurate Antioxidant Capacity Assessment of Honeys

**DOI:** 10.3390/mps9040120

**Published:** 2026-08-18

**Authors:** Ivan Lozada Lawag, Sharmin Sultana, Lee Yong Lim, Cornelia Locher

**Affiliations:** 1 Department of Pharmacy and Centre for Optimisation of Medicines, School of Health and Clinical Sciences, University of Western Australia, Crawley, Perth, WA 6009, Australia; sharmin.sultana@uwa.edu.au (S.S.); lee.lim@uwa.edu.au (L.Y.L.); 2 Institute of Herbal Medicine, National Institutes of Health, Department of Pharmacology and Toxicology, University of the Philippines Manila, 3rd Floor, Salcedo Hall, 547 Pedro Gil Street Corner A. Mabini Street, Ermita, Manila 1000, Philippines; 3 Institute for Paediatric Perioperative Excellence, University of Western Australia, Crawley, Perth, WA 6009, Australia

**Keywords:** ferric-reducing antioxidant power (FRAP) assay, honey, TPTZ, UV–Vis spectrophotometry, matrix effects, matrix-matched blanking, assay optimisation and validation, flavonoids, phenolic acids, iron–polyphenol interactions

## Abstract

This study optimised and validated a microplate Ferric-Reducing Antioxidant Power (FRAP) assay for honey that incorporates a honey-specific matrix-matched blank—a paired absorbance measurement lacking the chromogenic probe 2,4,6-tris(2-pyridyl)-s-triazine (TPTZ)—to separate true ferric reduction from non-reductive Fe^3+^–matrix interactions that otherwise inflate apparent antioxidant capacity. A 30 min incubation at 37 °C in the dark was selected as the optimal compromise between reaction completeness and reagent/matrix stability, using 20 µL sample and 180 µL FRAP reagent per well (absorbance at 620 nm). The assay was validated according to International Council for Harmonisation (ICH) principles, showing linearity for FeSO_4_·7H_2_O from 200–1200 µM (R^2^ ≥ 0.9988), Limit of detection/limit of quantification (LOD/LOQ) of 0.28/0.94 mmol Fe^2+^ equivalents kg^−1^, recoveries of 101–103%, and precision ≤ 5% relative standard deviation (RSD). All tested individual sugars except maltodextrin and the artificial honey matrix produced FRAP responses below the LOQ. Flavonoids, however, showed pronounced, time-dependent, non-reductive Fe^3+^ interactions and were markedly overestimated under conventional water blanking, while phenolic acids were less affected. Applied to 47 Western Australian honeys, the validated assay yielded FRAP activities of 2.80–9.52 mmol Fe^2+^ kg^−1^; matrix-matched blanking gave consistently lower, more chemically specific values than conventional water blanking, with corrections ranging up to >30% depending on honey type. Matrix-matched blanking is therefore essential, not optional, for accurate FRAP measurement in honey, and the same principle should apply directly to other phenolic-rich foods like wine, tea, and fruit extracts—wherever iron–polyphenol interactions confound conventional antioxidant assays.

## 1. Introduction

Honey is well known for its nutritional value and therapeutic properties, particularly its antioxidant activity [1,2], antimicrobial [3,4], anti-inflammatory [5,6,7], anti-aging [8,9,10], anticancer [11,12,13], and wound-healing effects [14,15,16]. The antioxidant potential of honey is linked to its diverse chemical composition, including flavonoids, phenolic acids, vitamins, enzymes, organic acids, and trace elements [1,17,18,19,20,21,22]. Phenolics and flavonoids are widely recognised as major contributors to honey’s antioxidant properties, owing to their capacity to participate in redox reactions and to modulate oxidative processes in biological systems [17,23,24,25]. Honey’s antioxidant capacity is influenced by multiple factors, including its geographical and botanical origins as well as processing and storage conditions, leading to substantial variability among samples [1,26,27]. This variability necessitates robust and standardised analytical methodologies for accurate, chemically meaningful assessment of honey’s antioxidant capacity [28,29].

**Figure 1:**
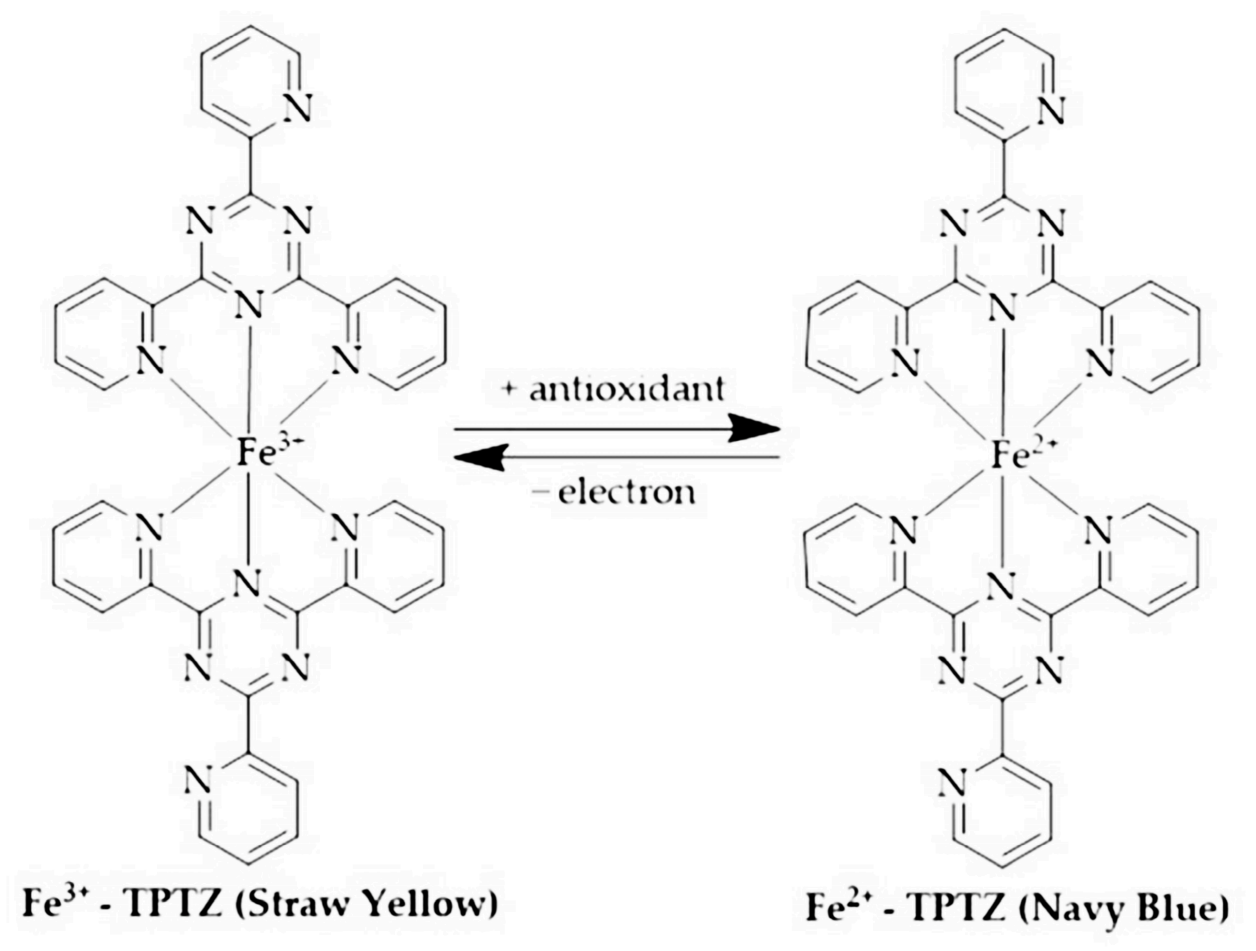
Schematic representation of the FRAP reaction mechanism. The ferric–2,4,6-tripyridyl-s-triazine complex (Fe^3+^–TPTZ; straw yellow) is reduced by antioxidants via single-electron transfer to form the ferrous complex (Fe^2+^–TPTZ; navy blue). This redox transformation under acidic conditions (pH ≈ 3.6) yields a stable chromophore with a strong absorbance at 593 nm, proportional to the sample’s ferric-reducing capacity. (Figure adapted from Xiao et al. [34] and Shi et al. [35]). For clarity, the single-electron-transfer step represented by the two arrows above can be summarised as: Fe^3+^–TPTZ + e^−^ (donated by antioxidant) → Fe^2+^–TPTZ; the reverse arrow denotes that this transfer is, in principle, reversible in the absence of a sustained electron donor.

A review of Scopus-indexed literature shows that the 2,2-diphenyl-1-picrylhydrazyl (DPPH) assay is the most frequently used method for honey (87%), followed by the FRAP assay (44%), the ABTS assay (23%), and ORAC (7%) [30]. Notably, 71% of studies employ at least two assays, reflecting the popularity of complementary measurements. While DPPH and ABTS assess radical-scavenging activity [31,32], FRAP measures reducing power [33] and its findings often correlate well with total phenolic content—a distinction this manuscript maintains throughout: FRAP does not measure “antioxidant activity” in the radical-quenching sense, but rather in the operationally defined capacity of a sample to reduce Fe^3+^. Specifically, the FRAP assay quantifies the reduction of Fe^3+^–TPTZ to the blue Fe^2+^–TPTZ complex at 593 nm (Figure 1), which results from rapid single-electron transfer [33]. Under typical acidic reaction conditions, FRAP measures an operationally defined ferric-reducing capacity, as it only captures compounds that rapidly reduce Fe^3+^ to form the Fe^2+^–TPTZ rather than accounting for all chemically reducing species present in the matrix.

Despite its wide application to foods [36], including honey, FRAP is prone to matrix effects and intrinsic methodological limitations [28,29,37,38,39,40], motivating refinement specifically for the honey matrix [28,29,39,40]. The assay is typically performed at pH 3.6 to maintain iron solubility, thereby imparting a hydrophilic bias in which water-soluble reductants dominate the response, and proteins may be underrepresented due to acid-induced denaturation and precipitation [37]. Lipophilic antioxidants such as α-tocopherol and carotenoids also respond weakly under these conditions, leading to systematic underestimation in matrices where they are present. Intrinsic colour can further confound readings, and Clarke et al. have addressed this limitation by introducing a colour control in which the chromogenic probe TPTZ is replaced with its solvent, 40 mM HCl, while all other components of the FRAP reagent are retained. As a control, this modified reagent is added to the sample extract, and the resulting absorbance—arising solely from intrinsic sample colour and non-reductive Fe^3+^ interactions—is then subtracted from the absorbance obtained with the FRAP working reagent for that specific extract. This paired subtraction removes matrix-derived optical contributions, ensuring that the reported FRAP signal reflects true Fe^3+^ reduction rather than background colour or metal–ligand complexation [29].

This paired-subtraction approach is what this study refers to throughout as matrix-matched blanking: the practice of subtracting, from each sample’s FRAP reading, an absorbance measurement taken from that same sample under identical reaction conditions but without TPTZ, so that only the signal attributable to true Fe^2+^–TPTZ formation remains. This term is defined here at first use and applied consistently throughout the manuscript (see Section 2.6 for the full protocol). Throughout, ‘matrix-matched blanking’ refers to this strategy/process, while ‘matrix-matched blank’ refers to the physical reagent, solution, or well to which it is applied (e.g., ‘a matrix-matched blank well’); the two terms are not used interchangeably.

Chemically, the FRAP assay involves electron transfer that reduces Fe^3+^–TPTZ to Fe^2+^–TPTZ (E°′ ≈ +0.70 V), so any sufficiently strong reductant can elevate the signal irrespective of its ability to facilitate radical quenching. Relatively weak reductants present at sufficiently high concentrations, Maillard products, and compounds that alter iron speciation can therefore yield apparent positives, whereas thiols and slow-reacting phenolics like quercetin and caffeic acid may be underrepresented at conventional incubation times [36,41,42].

**Figure 2:**
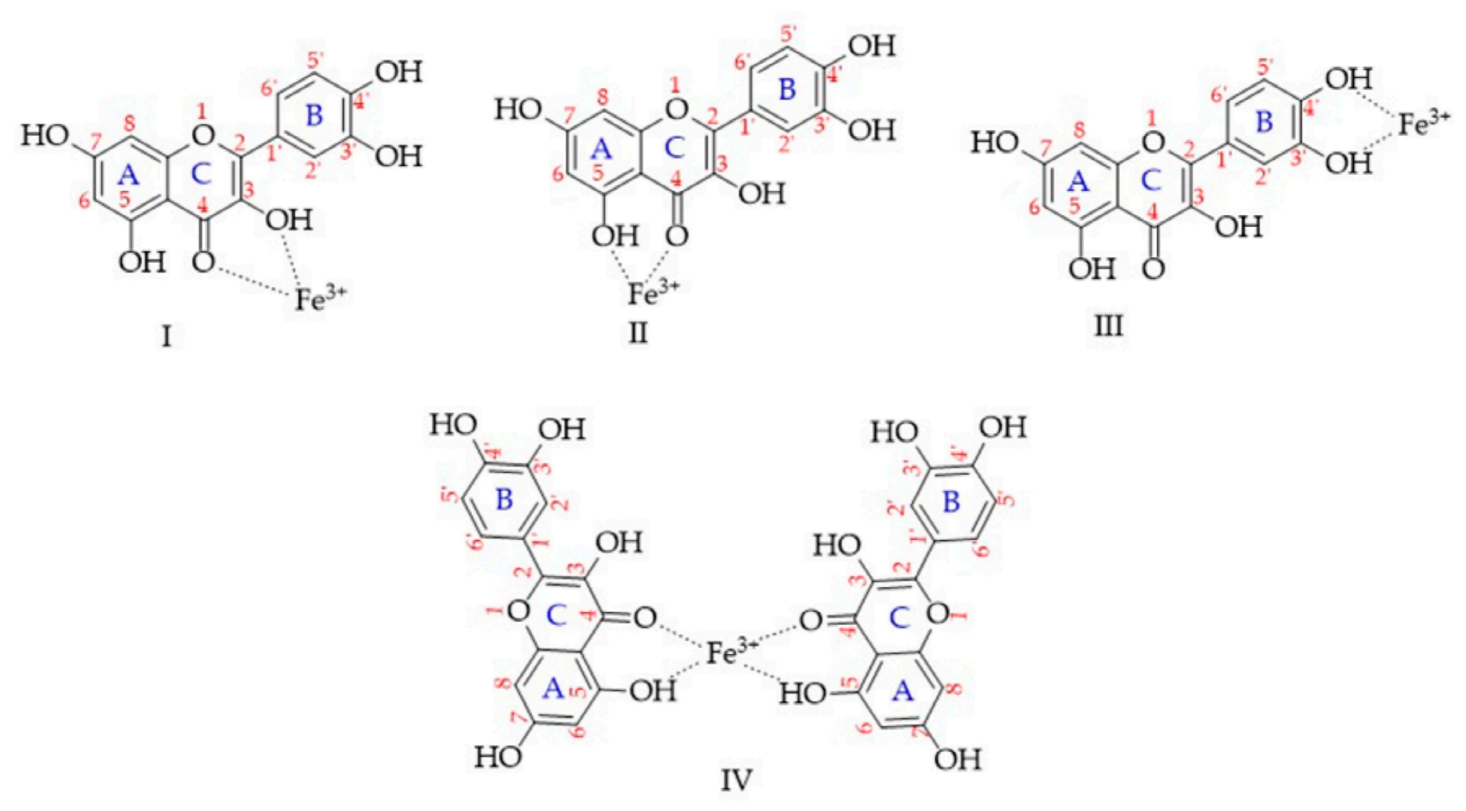
Proposed coordination modes between flavonoid structures and ferric iron (Fe^3+^). Panels I–III illustrate intramolecular chelation sites within a single flavonoid molecule; Panel IV depicts intermolecular bridging, where Fe^3+^ is coordinated simultaneously by oxygen donors from two separate flavonoid molecules, forming a bis-complex. (Figure adapted from Lesjak et al. [47]).

Moreover, the FRAP working solution contains FeCl_3_ in large excess, creating conditions that favour metal–ligand interactions. Many phenolics abundant in honey, particularly flavonoids such as quercetin and selected phenolic acids, possess catechol and 3-hydroxy-4-keto motifs that serve as high-affinity chelating sites for Fe^3+^. These structural features enable stable Fe^3+^–polyphenol complexation (Figure 2) [43,44], thereby modifying the system’s optical properties independently of ferric reduction. Under FRAP assay conditions, such complexes may contribute absorbance in the visible range that overlaps the analytical wavelength, thereby introducing non-specific signals unrelated to Fe^2+^–TPTZ formation [43,44,45,46]. Consequently, in phenolic-rich matrices such as honey, FRAP values may reflect a composite of rapid electron transfer to Fe^3+^ and the formation of metal–phenolic complexes, rather than a discrete measure of ferric-reducing capacity.

In honey, these methodological concerns have historically been attributed to the dominance of reducing sugars in its matrix. Glucose and fructose are the predominant sugars in honey [48], have been proposed as potential interferents in FRAP measurements. Early studies attributed part of the apparent antioxidant capacity of honey to reducing sugars, prompting the use of sugar analogues (‘artificial honey’) as matrix blanks [28]. However, the extent to which sugars contribute to the FRAP signal under acidic assay conditions—where ferric reduction, rather than overall reducing capacity, is measured—remains unclear and warrants systematic evaluation. This study, therefore, aimed to optimise and validate the FRAP assay specifically for honey by addressing matrix-dependent effects, which were historically attributed to reducing sugars, as well as non-specific phenolic interactions with excess Fe^3+^. By refining critical parameters, including incubation time and reagent stability, and by introducing a honey-specific matrix-matched blanking strategy, this work establishes assay conditions tailored to honey’s complex composition and provides a more accurate, analytically sound framework for measuring ferric-reducing capacity.

## 2. Materials and Methods

### 2.1. Literature Survey Strategy

A comprehensive literature search was conducted using the Scopus and Google Scholar databases, covering publications available up to March 2025. The keywords “Ferric-Reducing Antioxidant Power” and “honey” were used to identify studies that evaluated the antioxidant activity of honey using the FRAP assay. Articles were included if they met the following criteria: (1) published in English; (2) full text available; (3) employed 2,4,6-tripyridyl-s-triazine (TPTZ) as the Fe^3+^ probe; and (4) analysed honey in its natural form rather than as an additive or component of a formulated product. Review articles, conference proceedings, and retracted papers were excluded. In addition, publications authored by our research group were excluded to avoid bias.

From each eligible study, key experimental parameters relevant to FRAP-based honey analysis were extracted to enable systematic comparison. These included the volumes of FRAP reagent and honey sample used to determine whether a miniaturised format was adopted; honey concentration to assess the influence of dilution; incubation time and temperature, as these govern reaction kinetics and the completeness of Fe^3+^ reduction; detection wavelength; and the blanking strategy, because the choice of blank directly affects correction for non-antioxidant contributions. Preparation of the FRAP reagent was largely uniform across studies and was therefore omitted from the reporting table, allowing emphasis on variables most responsible for methodological divergence and inter-study variability.

### 2.2. Chemicals and Reagents

All reagents used in this study were of analytical grade, and all solutions were prepared using deionised water unless otherwise stated.

Fructose (CAS 57-48-7) and maltose (CAS 69-79-4) were obtained from Sigma-Aldrich (Truganina, Victoria, Australia). Mannose (CAS 3458-28-4), xylose (CAS 58-86-6), galactose (CAS 59-23-4), sucrose (CAS 57-50-1), glucose (CAS 50-99-7), maltodextrin (CAS 9050-36-6), and lactose (CAS 63-42-3) were supplied by Chem-Supply Pty Ltd. (Port Adelaide, South Australia, Australia). Methanol (CAS 67-56-1) was sourced from Scharlau (Barcelona, Spain), hydrochloric acid (CAS 7647-01-0) from Asia Pacific Specialty Chemicals Ltd. (Seven Hills, New South Wales, Australia), and anhydrous sodium acetate (CAS 127-09-3) and glacial acetic acid (CAS 64-19-7) from Ajax Chemicals Ltd. (Mt Pritchard, New South Wales, Australia).

2,4,6-Tris(2-pyridyl)-1,3,5-triazine (TPTZ; CAS 3682-35-7), iron (II) sulphate heptahydrate (FeSO_4_·7H_2_O; CAS 7782-63-0), and iron (III) chloride hexahydrate (FeCl_3_·6H_2_O; CAS 10025-77-1) were purchased from Sigma-Aldrich (Truganina, Victoria, Australia). Taxifolin (CAS 480-18-2) and naringin (CAS 10236-47-2) were obtained from AK Scientific, Inc. (Union City, CA, USA); epicatechin (CAS 490-46-0) from Wuhan ChemFaces Biochemical Co., Ltd. (Wuhan, Hubei, China); and gallic acid (CAS 149-91-7) from Ajax Finechem Pvt. Ltd. (Mt Pritchard, New South Wales, Australia).

Fisetin (CAS 528-48-3), pinobanksin (CAS 548-82-3), catechin (CAS 154-23-4), formononetin (CAS 485-72-3), genistin (CAS 529-59-9), and 2,3,4-trihydroxybenzoic acid (CAS 610-02-6) were purchased from Angene International Ltd. (Nanjing, China). Apigenin (CAS 520-36-5), chrysin (CAS 480-40-0), luteolin (CAS 491-70-3), myricetin (CAS 529-44-2), quercetin (CAS 117-39-5), hesperidin (CAS 520-26-3), naringenin (CAS 67604-48-2), epigallocatechin (CAS 970-74-1), genistein (CAS 446-72-0), protocatechuic acid (CAS 99-50-3), methyl syringate (CAS 884-35-5), syringic acid (CAS 530-57-4), trans-cinnamic acid (CAS 140-10-3), ferulic acid (CAS 537-98-4), rosmarinic acid (CAS 20283-92-5), and m-coumaric acid (CAS 14755-02-3) were obtained from Combi-Blocks Inc. (San Diego, CA, USA). Lumichrome (CAS 1086-80-2) and kojic acid (CAS 501-30-4) were sourced from Sigma-Aldrich (Castle Hill, New South Wales, Australia).

### 2.3. Honey Samples, Artificial Honey, and Sugar Solution Preparation

Table 1 provides details of the honey samples analysed in this study. Except for Manuka honey, which was sourced commercially, all samples were obtained from local beekeepers in Western Australia.

Optimisation of the FRAP assay was performed using the five Coastal Peppermint honey samples. Coastal Peppermint honey was selected as the matrix for method development because it was available in sufficient quantities and exhibited moderate and consistent antioxidant activity. Each honey sample was prepared in triplicate as a 20% (*w*/*v*) aqueous solution. This concentration was used throughout the optimisation experiments described in Section 2.5.1, Section 2.5.2 and Section 2.5.3 and in the final validated protocol described in Section 2.6 onward. Honey concentration was not evaluated as an optimisation variable; therefore, all honey-matrix method-development experiments, including the incubation-time findings presented in Section 3.2, were conducted using the 20% (*w*/*v*) honey matrix. Other validation experiments used the appropriate reference standards or control matrices described in the relevant sections. Subsequently, one of the Coastal Peppermint (*Agonis flexuosa*) honey samples was used as a positive honey control in all experimental runs to monitor assay reproducibility across multiple days.

Prior to sampling, each honey was gently warmed at 40 °C for 10 min in a Köttermann 3043 water bath (Köttermann GmbH & Co. KG, Uetze, Germany) and stirred to ensure homogeneity. An approximately 20% (*w*/*v*) aqueous honey solution was prepared by accurately weighing ~0.40 g of honey, recording the exact mass, and adjusting the final volume to 2.00 mL with deionised water. The mixture was vortex-homogenised using a D-LAB MX-S vortex mixer (DLAB Scientific Inc., Beijing, China) [49]. Prepared honey solutions were stored at −85 °C to arrest enzymatic activity and prevent oxidative or compositional changes prior to analysis.

Artificial honey was prepared by dissolving 21.625 g of fructose, 18.125 g of glucose, 1.000 g of maltose, and 0.750 g of sucrose in 8.500 g of water [50]. This mixture was treated in the same manner as natural honey samples, including warming at 40 °C for 10 min to ensure homogeneity. A 40% (*w*/*v*) aqueous artificial honey solution was then prepared in deionised water and vortex-mixed to achieve uniform consistency before analysis. The higher concentration (40% *w*/*v*) of artificial honey was used to spike ferrous standards, ensuring equivalent sugar loading between matrix-matched and water-based calibration solutions.

In parallel, individual 20% (*w*/*v*) aqueous sugar solutions of fructose, galactose, glucose, lactose, maltodextrin, maltose, mannose, sucrose, and xylose were prepared. These control solutions were used to evaluate assay specificity and the contribution of individual sugars to FRAP responses [51].

### 2.4. Reagent Preparation

A sodium acetate buffer was prepared by dissolving 3.100 g of anhydrous sodium acetate in 800 mL of deionised water, followed by the addition of 16.00 mL of glacial acetic acid. The pH was adjusted to 3.6 using hydrochloric acid (HCl) or sodium hydroxide (NaOH), and the final volume was brought to 1 L with deionised water. The buffer was stored at room temperature.

The 10 mM TPTZ reagent was prepared by dissolving 0.312 g of TPTZ in 40 mM HCl and making the solution up to 100 mL with 40 mM HCl. The solution was sonicated to ensure complete dissolution and stored at −85 °C to minimise degradation.

The ferric chloride reagent (20 mM) was prepared by dissolving 270.3 mg of FeCl_3_·6H_2_O in deionised water and adjusting the final volume to 50.0 mL. The solution was stored at −85 °C to preserve stability.

A 2 mM ferrous sulphate stock solution was prepared by dissolving 27.8 mg of FeSO_4_·7H_2_O in deionised water to a final volume of 50.0 mL. Serial dilutions were prepared to obtain working standards of 100, 200, 400, 600, 800, 960, 1000, 1200, and 1440 µM. The 960, 1200, and 1440 µM levels corresponded to 80%, 100%, and 120%, respectively, of the nominal working concentration used for accuracy assessment. A separate 600 µM FeSO_4_·7H_2_O solution was prepared and used as the positive control for the FRAP assay. All Fe^2+^ standards were prepared fresh before each experiment and stored on ice to minimise oxidation.

The FRAP working reagent was prepared fresh before each assay according to the original FRAP procedure [37,38] and approaches reported in honey and food antioxidant studies [52,53,54,55,56], with adaptations for honey analysis [57], by mixing sodium acetate buffer, TPTZ reagent, and FeCl_3_ solution in a 10:1:1 (*v*/*v*/*v*) ratio. The mixture was incubated at 37 °C in the dark for 30 min before use.

The FRAP blanking reagent was prepared in the same manner as the working reagent, except that the TPTZ solution was replaced with its solvent, 40 mM HCl, yielding a reagent containing acetate buffer and excess Fe^3+^ but no chromogenic probe. This acid-only blanking approach was adapted from the colour-control strategy described by Clarke et al. [29] and further developed in the present study for honey analysis. The reagent was freshly prepared before each assay and incubated at 37 °C in the dark for 30 min before use.

For phenolic and flavonoid testing, stock solutions of each compound were prepared at 0.5 mg/mL in methanol. These primary solutions served as intermediate stocks and were subsequently diluted with methanol to obtain working solutions at 7.813 µg/mL. A 20 µL aliquot of each working solution was added to 180 µL of FRAP reagent, yielding a final in-well concentration of 0.781 µg/mL. This concentration was selected based on preliminary testing to remain within the measurable response range of the phenolic panel while avoiding excessive signal saturation for strongly Fe^3+^-interacting flavonoids and maintaining measurable responses for less reactive phenolic acids. The phenolic standards included phenolic acids (syringic acid, rosmarinic acid, ferulic acid, *trans*-cinnamic acid, protocatechuic acid, gallic acid, 2,3,4-trihydroxybenzoic acid, methyl syringate, and *m*-coumaric acid), flavonoids (catechin, hesperidin, epicatechin, myricetin, quercetin, apigenin, chrysin, luteolin, fisetin, pinobanksin, naringenin, epigallocatechin, genistein, taxifolin, genistin, formononetin, and naringin), and other bioactive compounds (kojic acid and lumichrome). Stock solutions were stored at −85 °C to minimise oxidative degradation and maintain stability across experimental runs.

### 2.5. Optimisation of the FRAP Assay for Honey Analysis

#### 2.5.1. Reagent Stability Assessment

The baseline absorbance of the FRAP working reagent was monitored at 620 nm over 90 min under assay conditions to assess its stability in the absence of antioxidant reduction. In a 96-well microplate, 20 µL of deionised water was combined with 180 µL of freshly prepared FRAP working reagent. The plate was incubated at 37 °C in the dark, and absorbance (*A*_620_) was recorded at 4, 10, 15, 30, 45, 60, 75, and 90 min. Measurements were performed in triplicate wells within the same microplate, and results are reported as mean ± SD. No matched blank was applied, as the objective of this experiment was to assess the inherent stability of the reagent in the absence of any sample or matrix contribution. Data collected were evaluated by one-way repeated-measures ANOVA (*p* ≤ 0.05) with Tukey post hoc analysis. The FRAP working reagent was considered temporally stable when no statistically significant change in mean *A*_620_ was observed across the monitoring interval.

#### 2.5.2. Time-Dependent Absorbance Development in the Honey Matrix-Matched Blank

For Coastal Peppermint honey, a matrix-matched blank was prepared to quantify the matrix-derived background signal in the absence of TPTZ. In a 96-well microplate, 20 µL of honey solution was combined with 180 µL of FRAP blanking reagent. Plates were incubated at 37 °C in the dark, and absorbance at 620 nm (*A*_620_) was recorded at 4, 10, 15, 30, 45, 60, 75, and 90 min. Because TPTZ was omitted, these measurements represent only the honey-derived background arising from its intrinsic colour and other non-specific matrix contributions. For each time point, three analytical replicate wells (*n* = 3) were measured in a single experimental run using a single Coastal Peppermint honey sample; this is the same *n* = 3 replication structure used for the reagent stability assessment in Section 2.5.1. Results were expressed as mean ± SD. Temporal changes in background absorbance were evaluated using one-way repeated-measures ANOVA (*p* ≤ 0.05) with Tukey post hoc analysis. The matrix-matched blank was considered time-invariant when no statistically significant change in mean *A*_620_ was observed across the incubation period.

This experiment establishes whether the matrix background remains constant over the assay period. Demonstrating time invariance is essential for the validity of paired subtraction (i.e., matched-matrix blank absorbance subtracted from absorbance reading of sample), ensuring that ΔA (the change in absorbance after matrix blank subtraction) reflects only Fe^3+^–TPTZ reduction and not time-dependent matrix-derived artefacts.

#### 2.5.3. Optimisation of FRAP Assay Incubation Time

The time dependence of the FRAP signal development was evaluated using Coastal Peppermint honey (*n* = 4) at incubation times of 4, 10, 15, 30, 45, and 60 min using the matrix-matched blanking approach described above (Section 2.5.2). In a 96-well microplate, 20 µL of honey solution was combined with 180 µL of FRAP working reagent. Plates were incubated at 37 °C in the dark, and absorbance at 620 nm was recorded at each specified time point. This experiment was designed to characterise the temporal progression of FRAP signal formation and to identify a reaction window that accommodates both rapidly and slowly reacting antioxidant constituents while remaining within the reagent system’s temporal stability limits. The aim was not to define a universal kinetic optimum, as FRAP reaction kinetics may vary depending on sample composition, but to establish an analytically defensible incubation period anchored to the demonstrated stability window of the FRAP system for honey analysis. The incubation time adopted for the final assay protocol, therefore, corresponds to the longest interval that remained within the stability limits of both the FRAP working reagent and the matrix-matched blank. This strategy minimises underestimation from slowly reacting antioxidants while avoiding signal drift due to reagent or matrix instability, thereby providing a temporally robust framework for the assay.

### 2.6. Optimised FRAP Assay Protocol

**Figure 3:**
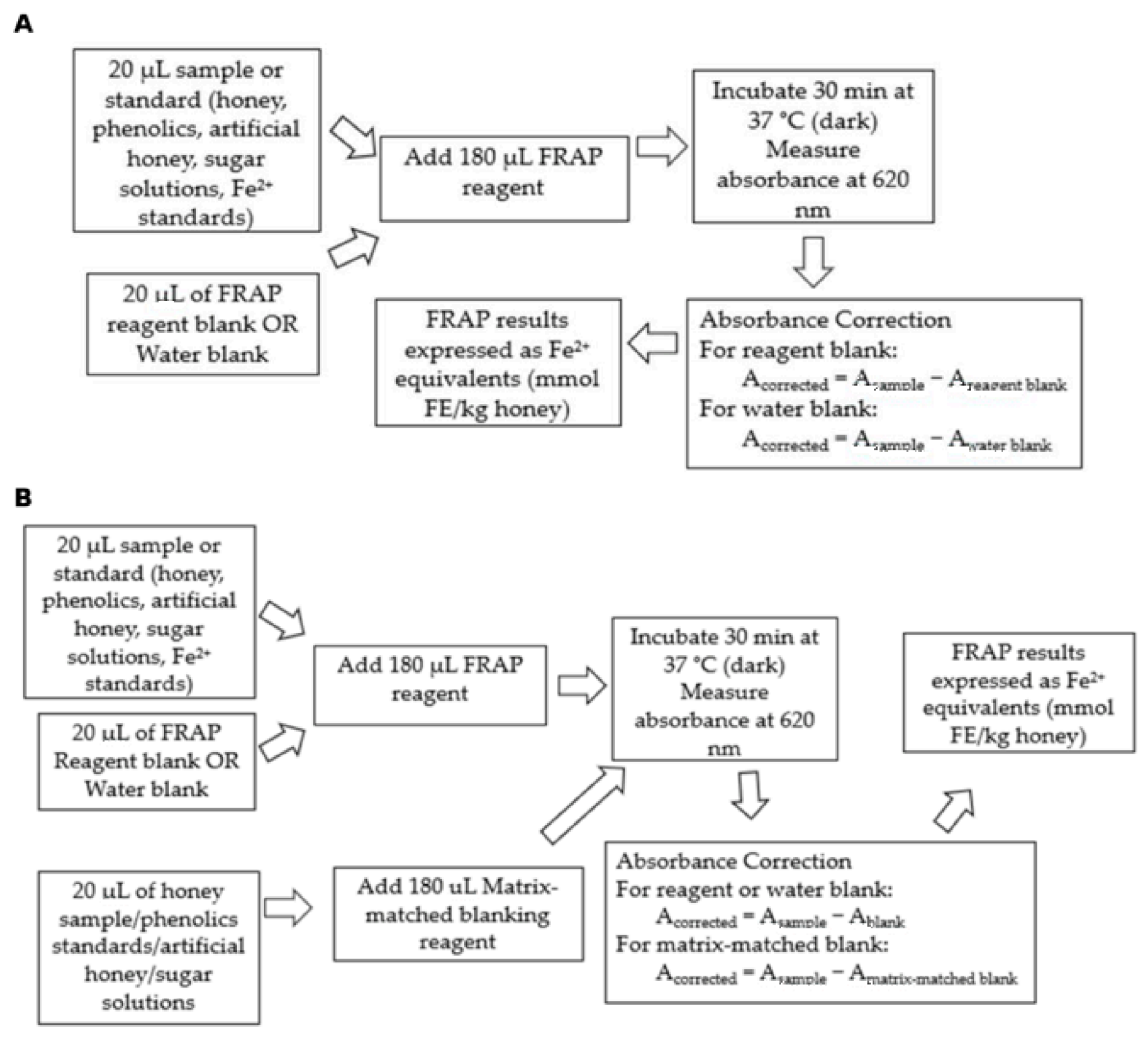
FRAP microplate protocols. (**A**) Conventional workflow using a FRAP-reagent or water blank. (**B**) Workflow incorporating the honey-specific matrix-matched blank developed in this study. Arrows indicate the direction of the experimental workflow; in (**B**), the two parallel streams (sample plus FRAP working reagent, and sample plus matrix-matched blanking reagent) converge at the incubation and absorbance-measurement step. Absorbance correction formulae for each approach are shown within the respective panel.

The FRAP assay was performed using a modified version of the method originally developed by Benzie and Strain [37] and later adapted for honey by Almeida et al. [57]. The conventional and matrix-matched blanking workflows used in the assay are illustrated in Figure 3. As mentioned earlier, the assay is based on the reduction of the ferric (Fe^3+^)–2,4,6-tripyridyl-s-triazine (TPTZ) complex to its ferrous (Fe^2+^) form under acidic conditions (pH 3.6), generating the intensely blue Fe^2+^–TPTZ chromophore. Although the typical detection wavelength is 593 nm [37], absorbance in this study was measured at 620 nm due to wavelength filter constraints of the microplate reader. This wavelength lies within the Fe^2+^–TPTZ absorbance band and remains proximal to the spectral maximum.

In the conventional FRAP assay, antioxidants in the sample reduce ferric ions bound to TPTZ by single-electron transfer, and the resulting absorbance is interpreted as total reducing capacity. Honey, however, is a chemically complex matrix containing not only various reductants but also numerous phenolic and non-phenolic constituents capable of interacting with ferric ions without necessarily reducing them. Such Fe^3+^–compound interactions can generate background absorbances that are indistinguishable from the true FRAP signal when only water or reagent blanks are used, leading to systematic overestimation of measured antioxidant capacity.

To resolve this limitation, a matrix-matched blanking strategy was implemented. In this approach, the chromogenic probe TPTZ is omitted and replaced by hydrochloric acid to prevent formation of the Fe^2+^–TPTZ complex while retaining excess Fe^3+^ in the reaction system. Under these conditions, any absorbance measured would arise solely from non-reductive interactions between Fe^3+^ and honey constituents. Subtraction of this sample-specific background from the absorbance reading of the corresponding FRAP reaction well isolates the absorbance attributable exclusively to electron-donating antioxidants. This matrix-matched blanking approach therefore distinguishes true ferric reduction from Fe^3+^ complexation artefacts and provides a chemically specific measure of reducing capacity in honey.

The protocol was optimised for miniaturised samples analysed in 96-well microplates to enable high-throughput screening. All honeys were analysed as 20% (*w*/*v*) aqueous solutions to standardise sample preparation and align with sample concentrations most reported in the literature. Assay parameters, including sample and reagent volumes, incubation temperature and duration, detection wavelength, standard selection, and result expression, were selected based on trends identified in the literature and refined experimentally to ensure analytical robustness and inter-study comparability. Incubation was conducted at 37 °C in the dark, and the reaction time was fixed at 30 min to accommodate both rapidly and slowly reacting antioxidant constituents while remaining within the stability window of the FRAP system. Ferrous sulphate was selected as the calibration standard because Fe^2+^ dissolves readily in water and avoids the acidification required for Trolox. Results were expressed as mmol Fe^2+^ equivalents per kilogram of sample, consistent with prevailing practice in FRAP-based honey studies.

For the assay, 20 µL of ferrous sulphate standards, honey samples, phenolic standards, artificial honey, or sugar solutions were dispensed into a 96-well flat-bottom microplate (Greiner Bio-One GmbH, Frickenhausen, Germany). One hundred eighty microliters (180 µL) of freshly prepared FRAP working reagent was added to each well, and the contents were mixed for 30 s using the microplate reader’s integrated mixing function. Plates were incubated in the dark at 37 °C for 30 min, after which absorbance was measured at 620 nm using a BMG Labtech POLARstar Optima microplate reader (BMG LABTECH GmbH, Ortenberg, Germany).

To correct for matrix-derived absorbance and non-specific reactions, matrix-matched blanking was applied to all honey samples, phenolic standards, artificial honey, and sugar solutions. For each analyte, a matched blank well was prepared using a FRAP blanking reagent identical to the working reagent but lacking TPTZ. These paired blanks were subjected to the same incubation and measurement conditions as their corresponding reaction wells. The net FRAP response was calculated as:

ΔA = *A*_620_ (reaction well) − *A*_620_ (matched sample blank) (1)

thereby isolating absorbance arising specifically from Fe^3+^ reduction. For Fe^2+^ calibration standards, correction was performed using water or FRAP-reagent blanks without a sample, as these solutions do not contribute intrinsic absorbance.

Antioxidant activity was quantified using a ferrous sulphate calibration curve and expressed as mmol Fe^2+^ equivalents (FE) per kilogram of honey, phenolic standard, artificial honey, or sugar solution. FRAP values were calculated according to:

FRAP Value of Sample (µM Fe(II)) = (ΔAbs − intercept)/slope(2)

where ΔAbs is the blank-corrected absorbance at 620 nm, and slope and intercept are derived from the FeSO_4_ calibration curve.

### 2.7. Validation of FRAP Assay Parameters for Honey Analysis

The validation process examined linearity and analytical range, specificity toward reducing compounds that facilitate rapid single-electron transfer, limits of detection (LOD) and quantification (LOQ), accuracy, and precision (repeatability and intermediate precision). Long-term assay performance was further evaluated using both a ferrous sulphate control and a representative honey matrix over 20 non-consecutive days to assess temporal stability under routine operating conditions.

#### 2.7.1. Linearity and Range

The linearity of the FRAP assay was initially assessed using a broad series of ferrous sulphate (FeSO_4_·7H_2_O) standards covering 100–1440 µM. This preliminary range was used to identify the concentration interval providing an acceptable linear response and precision under the optimised assay conditions. Standards were analysed in triplicate, in a randomised order, using the optimised FRAP protocol (Section 2.6). Absorbance was plotted against Fe^2+^ concentration and analysed by least-squares linear regression. The 100 µM level was excluded from the final validated range because its repeatability exceeded the predefined acceptance criterion of 5% RSD. Accordingly, the final validated linear range was established as 200–1200 µM. For accuracy assessment, 1200 µM was designated as the nominal working level (100%), while 960 and 1440 µM represented the 80% and 120% accuracy levels, respectively. The 1440 µM level was therefore used specifically for accuracy assessment and was not included in the final validated linear range. Linearity was evaluated using the coefficient of determination (R^2^) in accordance with ICH Q2(R2) principles.

#### 2.7.2. Limit of Detection (LOD) and Limit of Quantification (LOQ)

The limit of detection (LOD) and limit of quantification (LOQ) were calculated in accordance with the ICH Q2(R2) guidelines [51,58]. These parameters define the lowest concentration that can be reliably detected (LOD) and quantified (LOQ) with acceptable precision and accuracy, thereby ensuring that all reported sample measurements fall within the assay’s validated working range. LOD and LOQ were calculated using the following equations:

LOD = 3.3*σ*/*S* (3)

LOQ = 10*σ*/*S* (4)

where σ is the standard deviation of the response, and S is the slope of the calibration curve. The LOQ was additionally verified experimentally as the lowest calibration level meeting the predefined precision criterion (%RSD ≤ 5%).

#### 2.7.3. Specificity

The specificity of the FRAP assay for reducing compounds involved in rapid single-electron transfer was evaluated using the optimised protocol (Section 2.6) and by systematically examining the influence of sugar-rich matrices on the Fe^3+^–TPTZ reduction reaction. Ferrous sulphate (FeSO_4_·7H_2_O) standards were prepared in both deionised water and the artificial honey matrix to determine whether the presence of sugars alters the calibration response. In parallel, individual sugar solutions (fructose, galactose, glucose, maltose, lactose, maltodextrin, mannose, sucrose, and xylose) and artificial honey solutions were reacted with the FRAP reagent in the absence of FeSO_4_ to quantify their intrinsic reducing activity under assay conditions.

To assess whether a sugar-rich matrix influences the FRAP calibration response, two FeSO_4_·7H_2_O calibration series were prepared across the working range: (i) standards diluted in deionised water (unspiked standards) and (ii) standards diluted in artificial honey solution (0.4 g/mL; spiked standards) to provide a carbohydrate-rich calibration matrix. Each standard was analysed using the optimised FRAP microplate protocol, and absorbance at 620 nm was plotted against Fe^2+^ concentration to compare the calibration responses of spiked versus unspiked standards. To determine whether any calibration-matrix effect was propagated into sample quantification, Coastal Peppermint honey was analysed under the same assay conditions, and its FRAP activity was calculated twice: once using the unspiked (water-based) calibration curve and once using the artificial-honey-spiked calibration curve. In all of the above experiments, matrix effects were evaluated by comparing absorbance values and calculated FRAP equivalents obtained under water-blanked and matrix-matched conditions. Differences in absorbance and calculated FRAP values were analysed using a two-tailed *t*-test, with statistical significance defined as *p* < 0.05.

Phenolic compounds are known to interact with ferric ions through coordination and chelation, particularly via catechol and 3-hydroxy-4-keto motifs, forming Fe^3+^–polyphenol complexes that display characteristic optical responses in the visible region [59,60,61,62,63], including increased absorbance near the FRAP detection wavelength [60,63]. In the FRAP system, ferric ions are present in deliberate molar excess (20 mM FeCl_3_·6H_2_O in the working reagent) to ensure that reducing capacity is measured under Fe^3+^-non-limiting conditions. While this excess is essential for quantitative redox measurement, it also creates an environment in which phenolic compounds may interact with Fe^3+^ independently of electron transfer, generating background absorbance from interactions unrelated to Fe^3+^–TPTZ reduction activity.

To account for this non-reductive Fe^3+^ complexation, an alternative blanking system was evaluated by replacing the TPTZ probe with 40 mM HCl while retaining all other components of the FRAP reagent. The concentration of HCl was selected because 40 mM HCl is the solvent used to prepare the TPTZ reagent in the conventional FRAP system. Substituting TPTZ with 40 mM HCl while retaining all other components preserves the ionic strength, acidity, and chemical environment of the working reagent, ensuring that the only experimental difference between the reaction and blank wells is the absence of the chromogenic probe. This “FRAP blanking reagent” thus maintains the same excess Fe^3+^ concentration and conditions as the working reagent but cannot form the Fe^2+^–TPTZ chromophore. Any absorbance observed with this reagent reflects intrinsic sample colour and Fe^3+^–compound interactions rather than true ferric reduction.

The propensity of phenolic compounds to interact with excess FeCl_3_·6H_2_O under FRAP conditions was assessed using the optimised assay protocol (Section 2.6). Individual phenolic standards were reacted with the FRAP blanking reagent, and absorbance at 620 nm was recorded at time 0 and after 30 min of incubation at 37 °C in the dark. The same phenolic solutions were then analysed in parallel using the FRAP working reagent to generate conventional FRAP responses. For each compound, FRAP activity was calculated using two blanking strategies: (i) water blanking and (ii) paired-sample blanking, in which the matched FRAP blanking reagent served as the blank. The difference between water-blanked and sample-blanked FRAP values was used to quantify the magnitude of non-reductive interference attributable to Fe^3+^–compound interactions. A panel of flavonoids, phenolic acids, and non-phenolic bioactive compounds was examined and grouped by chemical class.

#### 2.7.4. Accuracy

The accuracy of the optimised FRAP assay was evaluated by analysing ferrous sulphate at three concentration levels corresponding to 80% (960 µM), 100% (1200 µM), and 120% (1440 µM) of the nominal working level [51]. Each concentration was prepared independently and analysed in triplicate using the optimised FRAP protocol (Section 2.6). Accuracy was expressed as percent recovery using the following equation:

% Recovery = (*C*_m_/*C*_t_) × 100 (5)

where Cm is the mean concentration determined from the calibration curve by interpolation, and Ct is the theoretical concentration of the test solution. The method was considered accurate when the percent recovery at each level was within 95–105%, and the relative standard deviation (RSD) did not exceed 5%.

#### 2.7.5. Precision and Long-Term Repeatability

The precision of the FRAP assay was assessed using repeatability (intra-day precision) and intermediate precision (inter-day precision) [51,58]. Intra-day precision was assessed by analysing triplicate standard solutions of FeSO_4_·7H_2_O at three concentration levels (80%, 100%, and 120% of the nominal working level) within a single analytical run. Intermediate precision was determined by repeating the same measurements over three separate days using freshly prepared reagents and standards.

Long-term repeatability was assessed using two controls: (i) a defined positive control consisting of 600 µM FeSO_4_·7H_2_O and (ii) a representative honey matrix (Coastal Peppermint honey). Each control was analysed in triplicate (*n* = 3) on 20 non-consecutive days using the optimised FRAP protocol. For intra-day and inter-day precision using FeSO_4_·7H_2_O standards, the assay was considered acceptable when the RSD at each concentration level did not exceed 5.0%. For long-term repeatability, an RSD of ≤5.0% was likewise considered acceptable, reflecting the greater variability expected under repeated measurements over an extended period and in a complex honey matrix.

### 2.8. Application of the Optimised and Validated FRAP Assay to Honey Analysis

All honey samples were analysed using the optimised FRAP protocol described in Section 2.6. Each honey sample was evaluated using two blanking approaches: conventional water blanking and paired-sample blanking using the FRAP blanking reagent. For paired blanking, two wells were prepared per honey. The reaction well contained 20 µL of honey solution and 180 µL of FRAP working reagent. The matched blank well contained 20 µL of the same honey solution and 180 µL of FRAP blanking reagent. Both wells were incubated at 37 °C in the dark for 30 min, and absorbance was measured at 620 nm. The net FRAP response was calculated as ΔA = *A*_620_ (reaction) − *A*_620_ (paired blank), yielding a signal attributable specifically to Fe^3+^ reduction. In parallel, FRAP values were also calculated using water blanking to obtain conventionally blanked results.

### 2.9. Statistical Analysis

Antioxidant activity calculations and linear regression modelling were performed using Microsoft Excel for Microsoft 365, Version 2607 (Build 20228.20190; Current Channel; Click-to-Run), Microsoft Corporation, Redmond, WA, USA. Statistical analyses were conducted using GraphPad Prism version 9.5.0 (GraphPad Software, San Diego, CA, USA). Depending on the experimental design, data were analysed using independent or paired *t*-tests, one-way analysis of variance (ANOVA), repeated-measures ANOVA, and Tukey’s post hoc paired comparisons. Data are presented as mean ± standard deviation unless otherwise stated. A *p*-value of < 0.05 was considered statistically significant.

### 2.10. Statement on the Use of AI-Assisted Tools

AI-assisted tools, including ChatGPT (OpenAI; GPT-5.6 Luna), Claude (Anthropic; Claude Opus 5), and Grammarly (v1.2.286.1939), were used during manuscript preparation for language refinement, grammar correction, paraphrasing, improving readability, sentence restructuring, organisation of the scientific discussion, and refinement of wording related to the interpretation of the experimental findings, under the supervision of the authors. All experimental design, data analysis, validation, interpretation, and scientific conclusions were independently reviewed and verified by the authors.

## 3. Results and Discussion

### 3.1. Literature Survey Findings

A total of 136 research articles met the inclusion criteria [2,3,4,5,6,28,52,53,54,55,64,65,66,67,68,69,70,71,72,73,74,75,76,77,78,79,80,81,82,83,84,85,86,87,88,89,90,91,92,93,94,95,96,97,98,99,100,101,102,103,104,105,106,107,108,109,110,111,112,113,114,115,116,117,118,119,120,121,122,123,124,125,126,127,128,129,130,131,132,133,134,135,136,137,138,139,140,141,142,143,144,145,146,147,148,149,150,151,152,153,154,155,156,157,158,159,160,161,162,163,164,165,166,167,168,169,170,171,172,173,174,175,176,177,178,179,180,181,182,183,184,185,186,187,188,189]. Full per-study data (FRAP assay parameters, blanking systems, calibration standards, and result expressions for all 136 studies) are provided in Supplementary Table S1; a condensed summary of the key trends is given in Table 2 below. In most cases, FRAP was implemented as an in-house laboratory protocol; only two research groups employed a commercially available FRAP kit [67,68]. Except for a single study that substituted the conventional acetate buffer with formic acid [68], the FRAP working solution was prepared according to the well-established protocol, comprising 300 mM acetate buffer (pH 3.6), 10 mM TPTZ in 40 mM HCl, and 20 mM FeCl_3_·6H_2_O in water.

Collectively, this methodological heterogeneity, particularly in incubation time, temperature, and light control, underscores the need for systematic optimisation and validation of FRAP conditions specifically for honey matrices.

More recently, acid-only blanks have been introduced for non-honey, plant-based FRAP assays, in which TPTZ is replaced with 40 mM hydrochloric acid to prevent the formation of the Fe^2+^–TPTZ chromophore while retaining excess Fe^3+^. This approach enables the subtraction of signals arising from intrinsic sample colour and non-reductive Fe^3+^–phenolic complexation [29]. However, this strategy has not been systematically evaluated in honey matrices, despite their high phenolic complexity. Its application in this study, therefore, represents a methodological precedent. Trolox, although widely used as a reference antioxidant, exhibits limited aqueous solubility and typically requires acidification for dissolution, introducing additional preparative variability. In contrast, ferrous salts dissolve readily in water, simplifying calibration and improving reproducibility. Consequently, 12% of studies report FRAP results as ferrous equivalents, most expressed as mmol Fe^2+^ equivalents per kilogram of honey.

### 3.2. Optimisation of FRAP Assay Conditions

#### 3.2.1. Reagent Stability Findings

**Figure 4:**
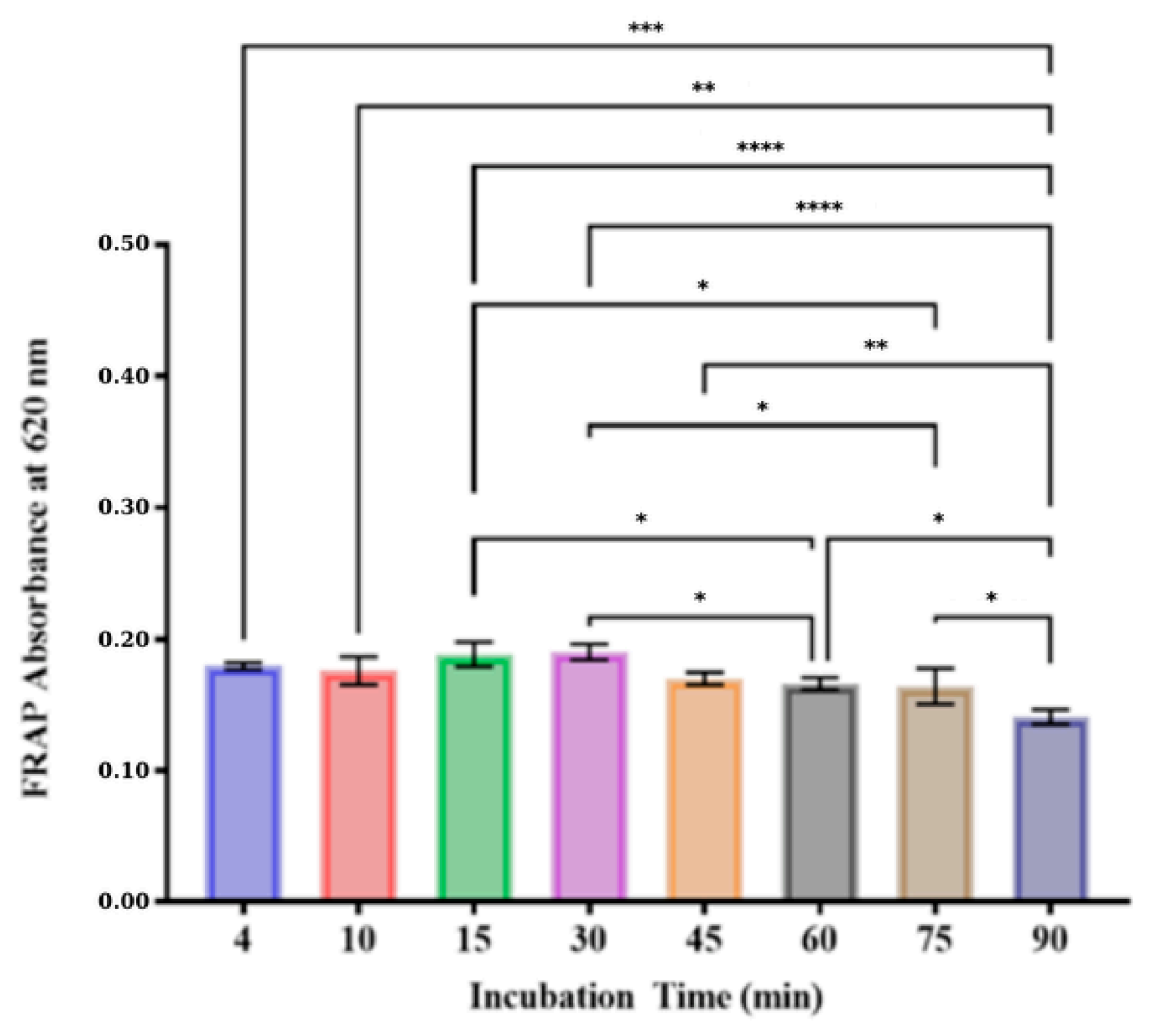
Absorbance of the FRAP working reagent stored in the dark at 37 °C, measured at different time points (*n* = 3). Data were analysed using a repeated-measures one-way ANOVA, followed by Tukey’s post hoc paired comparisons; only significant comparisons are shown. * *p* < 0.05, ** *p* < 0.01, *** *p* < 0.001, **** *p* < 0.0001.

Reagent stability testing revealed a time-dependent decline in intrinsic absorbance of the FRAP working reagent under assay conditions (Figure 4). The reagent was incubated at 37 °C in the dark to replicate the exact thermal environment commonly used during routine sample analysis. This step is analytically necessary because the FRAP reaction is strongly temperature-dependent: both the formation of the Fe^3+^–TPTZ complex and subsequent electron-transfer reactions proceed at markedly different rates at ambient temperature than at 37 °C [37,190]. Pre-equilibration at 37 °C ensures that the reagent and samples reach thermal equilibrium before measurement, thereby minimising variability arising from temperature-driven differences in reaction kinetics between wells and across plates.

Absorbance values were monitored over a 90 min period (*n* = 3 per time point). Repeated-measures one-way ANOVA followed by Tukey’s post hoc paired comparisons demonstrated significant reductions in reagent absorbance beginning after 30 min, with marked decreases between 30 and 45 min (*p* = 0.0152) and between 30 and 60 min (*p* = 0.0089). The decline became progressively more pronounced at 60, 75, and 90 min relative to the initial 4 min measurement (*p* ≤ 0.0004). In contrast, no significant differences were observed among the early time points (4, 10, 15, and 30 min; *p* > 0.05), indicating that the FRAP reagent remains spectrally stable within this window under assay conditions.

This time-dependent drift of the FRAP reagent has important analytical implications, particularly in microplate formats where sequential dispensing, incubation, and reading can introduce systematic bias across wells and plates [46]. Nevertheless, when the assay workflow is controlled so that dispensing, incubation, and measurement occur within the demonstrated stability window, the microplate format remains well-suited for high-throughput FRAP analysis of honey samples.

Honey is a matrix whose antioxidant profile includes phenolics and Maillard-derived compounds that exhibit heterogeneous, sometimes delayed, redox kinetics [28,40]. Short incubation times, although minimising reagent drift, risk underestimating these slowly reacting constituents. Conversely, extended incubation risks conflating true ferric reduction with reagent instability. The present datasets, therefore, justify selecting a 30 min reaction window as a compromise between reagent stability and analytical completeness. Accordingly, a fixed incubation time of 30 min was adopted for all subsequent assays.

#### 3.2.2. Time-Dependent Absorbance Findings in the Honey Matrix-Matched Blank

**Figure 5:**
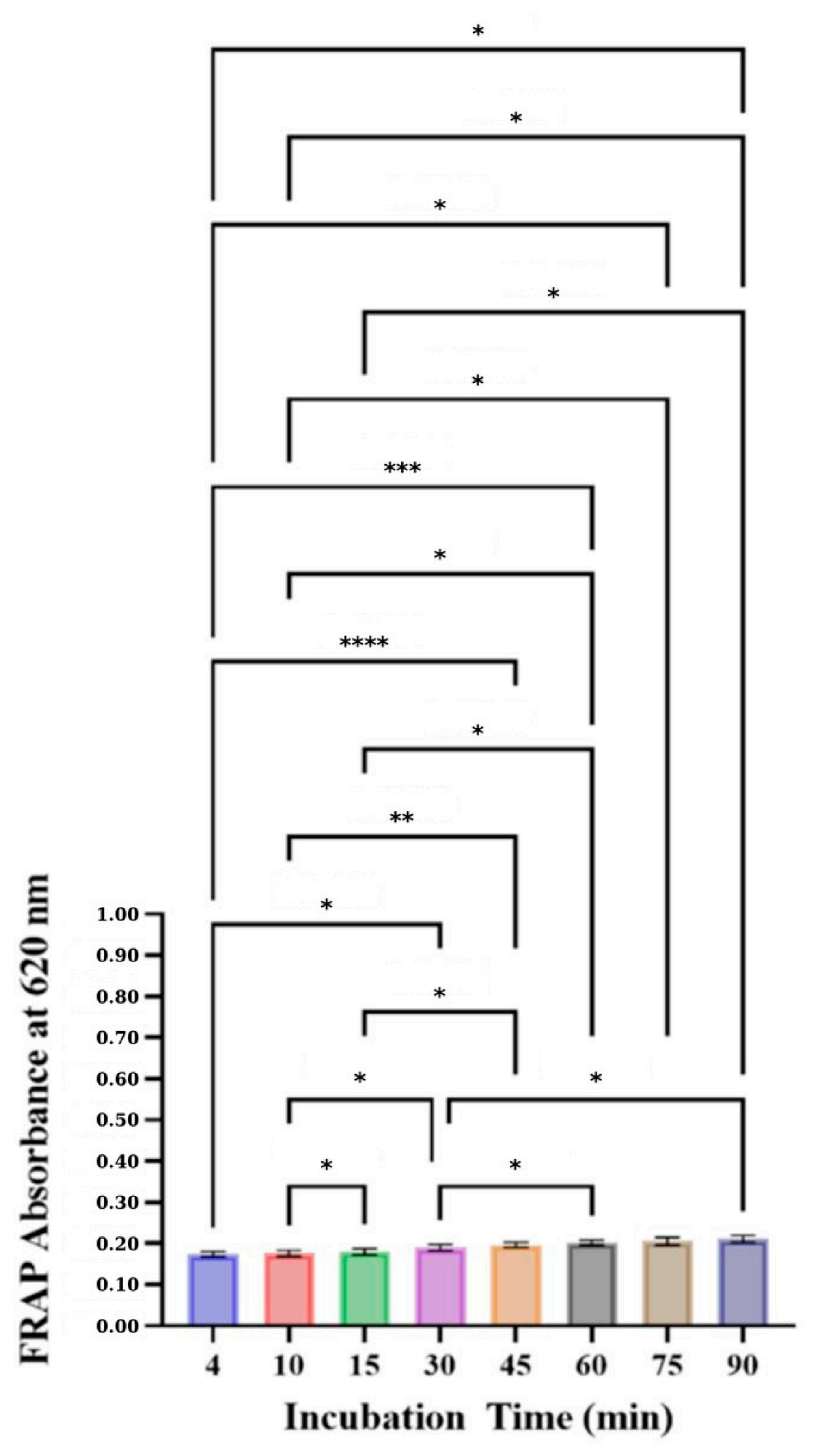
Absorbance profile of the Coastal Peppermint honey matrix-matched blank during incubation at 37 °C (*n* = 3). The blank consisted of honey mixed with acetate buffer, HCl and FeCl_3_, but without TPTZ. Data were analysed by repeated-measures one-way ANOVA followed by Tukey’s post hoc paired comparisons; only significant comparisons are shown. * *p* < 0.05, ** *p* < 0.01, *** *p* < 0.001, **** *p* < 0.0001.

Figure 5 presents the absorbance profile of the matrix-matched blank prepared from Coastal Peppermint honey during incubation at 37 °C in the dark for up to 90 min (*n* = 3 per time point). The matrix-matched blank solution consisted of the honey sample mixed with acetate buffer, HCl, and excess FeCl_3_, but without TPTZ. This composition reproduces the iron environment of the FRAP assay while preventing formation of the Fe^2+^–TPTZ chromophore. Consequently, any absorbance observed at 620 nm arises from optical contributions of the honey matrix in the presence of Fe^3+^ rather than from the FRAP redox reaction itself.

Absorbance increased progressively with incubation time. Repeated-measures one-way ANOVA followed by Tukey’s post hoc paired comparisons showed no statistically significant differences among the earliest time points (4, 10, and 15 min; *p* > 0.05). However, significant increases were observed from 30 min onwards relative to earlier intervals, with *p*-values ranging from 0.0458 (4 vs. 30 min) to <0.0001 for later comparisons.

These findings align closely with previous reports showing that phenolic compounds interact with Fe^3+^ ions through coordination and chelation, particularly via catechol and 3-hydroxy-4-keto motifs, forming Fe^3+^–polyphenol complexes with characteristic absorbance in the visible region, often near 593 nm [44,45,59,60,61,62,63]. The mixture was incubated at [37]. Such metal–phenolic species elevate background signals in the FRAP assay independently of true redox activity and are therefore indistinguishable from genuine FRAP responses when only water or reagent blanks are used. The observed time-dependent increase in the honey blank thus provides direct experimental evidence that non-reductive Fe^3+^–matrix interactions can confound FRAP measurements in honey.

Together with the reagent stability data (Section 3.2.1), these results demonstrate that both the FRAP reagent and the honey matrix exhibit intrinsic temporal behaviour under assay conditions. The coincidence of reagent stability up to 30 min and the onset of significant matrix drift beyond this interval provides a mechanistic justification for fixing the reaction time at 30 min. More importantly, the findings establish the necessity of paired, matrix-matched blanking in honey FRAP analysis to ensure that measured signals reflect genuine ferric reduction rather than artefacts arising from time-dependent Fe^3+^–phenolic complex formation [29].

#### 3.2.3. Optimisation of Incubation Time for FRAP Activity in Honey

In complex matrices such as honey, the FRAP assay reflects the cumulative reduction of the Fe^3+^–TPTZ complex by multiple antioxidants with different reaction kinetics. As originally described by Benzie and Strain (1996), absorbance increases progressively during the reaction window, particularly in samples containing phenolic compounds that react more slowly than rapidly reducing species such as ascorbate [37,42,46].

**Figure 6:**
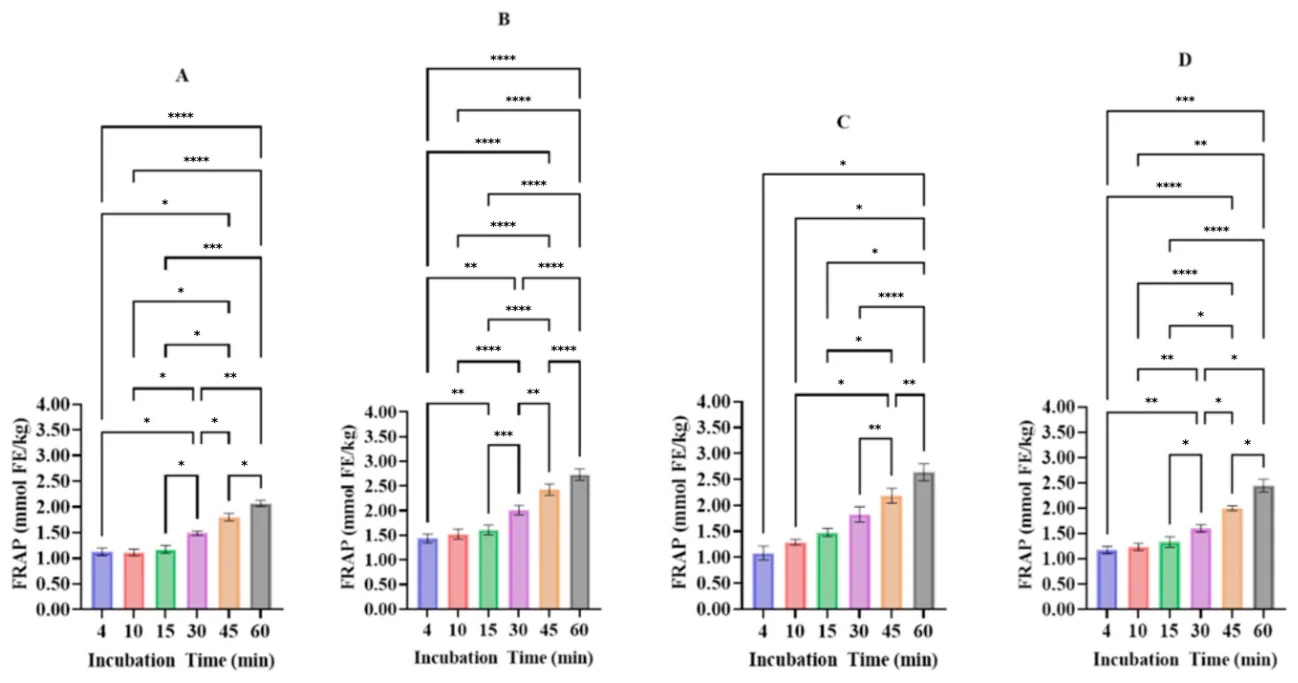
FRAP activity of Coastal Peppermint honey at varying incubation times. Panels (**A**–**D**) correspond to the four individual honey samples used in this experiment (*n* = 3 analytical replicates per sample per time point). Data were analysed by repeated-measures one-way ANOVA followed by Tukey’s post hoc paired comparisons; only significant comparisons are shown. * *p* < 0.05, ** *p* < 0.01, *** *p* < 0.001, **** *p* < 0.0001.

Consistent with this principle, Figure 6 shows a clear incubation-time dependence for Coastal Peppermint honeys analysed using matrix-matched blanking. Panels A–D each show one of the four individually sourced Coastal Peppermint honey samples used for this experiment (Section 2.5.3, *n* = 4 honeys), each assayed in analytical triplicate. No significant differences were observed across 4, 10, and 15 min (*p* > 0.05), whereas FRAP values increased significantly from 30 min onward, with further increments at 45 and 60 min across all four samples. The consistently higher values at 60 min indicate that a substantial fraction of honey antioxidants reacts slowly with the FRAP reagent, which is characteristic of phenolic-rich matrices [42,46].

However, the time-dependent increases in Figure 6 must be interpreted within the chemical stability constraints of the assay system. The stability experiments in Section 3.2.1 and Section 3.2.2 established that both the FRAP working reagent and the honey blanking solution remain stable for approximately 30 min. The kinetic experiment reported here was conducted separately, to characterize the evolution of the FRAP signal over time and to identify an incubation period that captures the major phase of Fe^3+^–TPTZ reduction. Although absorbance continues to increase beyond 30 min, both the reagent and blank solutions exhibit measurable drift after this point. Signal growth beyond the validated stability window may therefore no longer reflect exclusively antioxidant-driven Fe^3+^ reduction [43,46].

Because honey composition varies widely with botanical origin, the kinetic pattern observed in Figure 6 cannot be assumed to apply universally. An incubation time of 30 min, therefore, represents a conservative and chemically defensible compromise: it captures substantially more antioxidant activity than shorter intervals while remaining within the stability window of both the FRAP working reagent and the matrix-matched blank. Accordingly, 30 min was selected for all subsequent analyses, and FRAP values are reported as standardized operational measures of reducing capacity under controlled conditions rather than absolute kinetic endpoints [37,46].

#### 3.2.4. Optimised Conditions of the FRAP Assay for Honey Analysis

The optimised FRAP assay employed a reaction mixture consisting of 20 µL of honey or standard solution combined with 180 µL of FRAP reagent, yielding a total reaction volume of 200 µL per well. Incubation was performed for 30 min in the dark, balancing the need to allow sufficient time for both rapidly and slowly reacting antioxidant constituents to contribute to the signal with the requirement to remain within the temporal window during which both the FRAP reagent and the honey matrix remain chemically stable.

A honey-specific matrix-matched blanking strategy was incorporated to correct for background absorbance arising from non-reductive interactions between Fe^3+^ and honey constituents, particularly phenolic compounds capable of forming Fe^3+^ complexes. For each honey sample, a corresponding matrix blank was prepared using the same honey solution with TPTZ omitted. Conventional water or reagent blanking does not account for these matrix-driven optical contributions, which can inflate apparent reducing capacity. The matrix-matched blank, therefore, isolates absorbance attributable specifically to Fe^3+^ reduction, improving the chemical specificity of the assay.

### 3.3. Results of Validation

#### 3.3.1. Linearity and Range Findings

The linearity of the FRAP assay was assessed using ferrous sulphate standards ranging from 200 to 1200 µM. Each concentration level was analysed in triplicate, and repeatability at each level met the predefined acceptance criterion (%RSD ≤ 5%). Initial testing included a 100 µM standard; however, precision at this level exceeded the predefined 5% RSD acceptance threshold and it was therefore excluded from subsequent analyses.

Across three independent calibration runs, the optimised range (200–1200 µM) produced robust calibration curves with coefficients of determination (R^2^) of 0.9993, 0.9998, and 0.9988, exceeding the minimum linearity criterion (R^2^ ≥ 0.99). In addition, the consistency of slope and intercept values across these independent runs supports the intermediate precision (between-run reproducibility) of the calibration model.

#### 3.3.2. Limit of Detection (LOD) and Limit of Quantification (LOQ) Findings

The analytical sensitivity of the optimized FRAP assay was evaluated by determining the LOD and LOQ from the ferrous sulphate calibration data using the standard deviation of the response and the slope of the calibration curve, in accordance with ICH Q2(R2) principles. The method exhibited an LOD of 0.28 mmol Fe^2+^ equivalents per kilogram and an LOQ of 0.94 mmol Fe^2+^ equivalents per kilogram.

Direct comparison with literature values is limited, as LOD and LOQ are rarely reported in FRAP studies of honey. For example, Bolanos de la Torre et al. (2015) reported a minimum detectable concentration (~2.1 × 10^−7^ M) for a microplate FRAP assay but did not determine LOD or LOQ using validation-based approaches [187]. The LOQ obtained in the present study, therefore, represents a more formally defined sensitivity parameter and falls within the lower range of FRAP values typically reported for honey.

#### 3.3.3. Specificity Findings

The specificity of the FRAP assay was assessed to determine whether the high sugar content of honey interferes with reducing-capacity measurements. This evaluation was necessary because sugars can constitute up to approximately 80% of honey by mass [48] and therefore represent the predominant components of the honey matrix. All tested individual sugars, except maltodextrin, produced FRAP responses below the limit of quantification (LOQ). The 20% (m/v) artificial honey matrix also produced a response below the LOQ (Table 3). This supports the interpretation that, under the specified acidic assay conditions and 30 min incubation period, the FRAP signal is dominated by species capable of sufficiently rapid electron transfer to Fe^3+^–TPTZ rather than by the major reducing sugars present in honey

**Figure 7:**
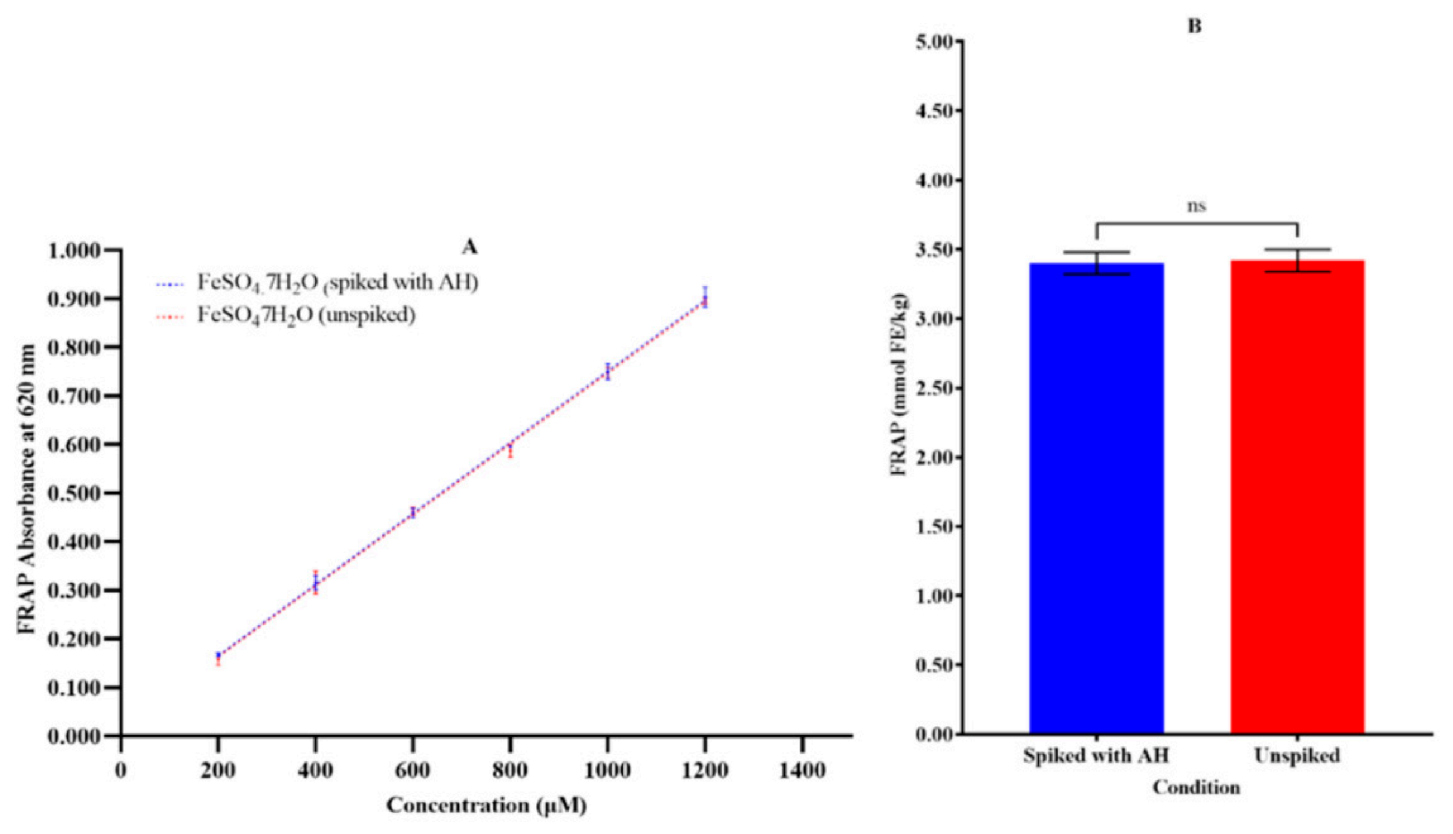
(**A**) Absorbance of standards spiked with Artificial Honey (blue) compared to unspiked standards (red). (**B**) FRAP activity of Coastal Peppermint honey, determined using standards spiked with Artificial Honey (blue) versus unspiked standards (red); ns = not significant (*p* ≥ 0.05, two-tailed *t*-test).

Comparative analysis of ferrous sulphate standards prepared in water and in the presence of artificial honey (20% *w*/*v*) showed highly consistent absorbance responses across the entire concentration range of 200–1200 µM (Figure 7A). Absorbance values at corresponding concentrations were compared using an independent samples *t*-test, and no significant differences were observed between the two media (*p* ≥ 0.05). Similarly, Coastal Peppermint honey samples analysed with and without spiking with artificial honey exhibited no significant differences in measured antioxidant activity, with mean values of approximately 3.4 mmol Fe^2+^ equivalents per kilogram in both cases (Figure 7B; *p* = 0.7309).

These results demonstrate that, at concentrations representative of natural honey, sugars do not introduce a statistically detectable bias in the measured FRAP response. This supports the interpretation that the FRAP signal is primarily governed by antioxidant species capable of rapid single-electron transfer, rather than by the slowly reacting reducing sugars that dominate honey’s carbohydrate composition.

Maltodextrin was the sole exception, exhibiting measurable FRAP activity (1.09 ± 0.06 mmol Fe^2+^ equivalents per kilogram). This is worth emphasising: maltodextrin is not a natural constituent of honey—its presence is specifically associated with adulteration or processing additives, so this modest reducing activity does not represent a naturally relevant contributor to the reducing capacity of authentic honey and is unlikely to influence antioxidant quantification in genuine samples. Its inclusion here instead serves as a worst-case, non-native carbohydrate control, showing that even a more reactive, partially hydrolysed sugar contributes only modestly relative to typical honey antioxidant values.

Phenolic antioxidants can contribute to apparent FRAP signals through two parallel processes: (i) true electron transfer that reduces Fe^3+^ to Fe^2+^ (the intended FRAP response), and (ii) non-reductive coordination/chelation of Fe^3+^ to form Fe^3+^–polyphenol complexes that can absorb in the visible region and distort absorbance-based readouts [43,44].

As summarized in Table 4 (full 28-compound dataset in Supplementary Table S2; three representative compounds shown below), flavonoids consistently showed the strongest evidence of Fe^3+^ interaction under FRAP conditions. Most flavonoids exhibited large and statistically significant increases in absorbance between 0 and 30 min in the Fe^3+^-containing blanking reagent, indicating time-dependent formation of Fe^3+^–flavonoid species. This behaviour is chemically plausible because many flavonoids contain structural motifs that bind Fe^3+^ strongly, including catechol groups on the B-ring and the 3-hydroxy-4-keto/4-keto-5-hydroxy coordination sites associated with flavonols [44,195,196]. In contrast, phenolic acids generally showed smaller absorbance changes in the Fe^3+^ blanking reagent and much smaller differences between water-blanked and matched-blanked FRAP values.

Overall, the full dataset demonstrates that non-reductive Fe^3+^–compound interactions are pronounced for many flavonoids, comparatively limited for most phenolic acids, and dependent on functional-group architecture. This flavonoid-versus-phenolic-acid distinction is the central specificity finding of this study: it is the reason a conventional water blank is inadequate for flavonoid-rich matrices such as honey, and the reason matrix-matched blanking (Section 2.6) was developed and validated here. These findings justify the use of matched blanking (Fe^3+^ present, no TPTZ) when applying the FRAP assay to honey or phenolic-rich matrices [43,44].

#### 3.3.4. Accuracy and Precision Findings

The results of the accuracy and precision tests for the FRAP assay are summarized in Table 5. Percentage recoveries ranged from 100.75% to 102.70%, falling well within the commonly accepted acceptance criterion of 95–105% for quantitative analytical methods, thereby confirming acceptable accuracy in accordance with ICH Q2(R2) principles. In terms of precision, intra-day (repeatability) %RSD values ranged from 1.98% to 2.90%, while inter-day (intermediate precision) %RSD values ranged from 1.50% to 2.82%. All values were well below the 5% threshold generally accepted for analytical precision under ICH validation guidance. These data demonstrate excellent repeatability and reproducibility of the optimised FRAP assay.

**Figure 8:**
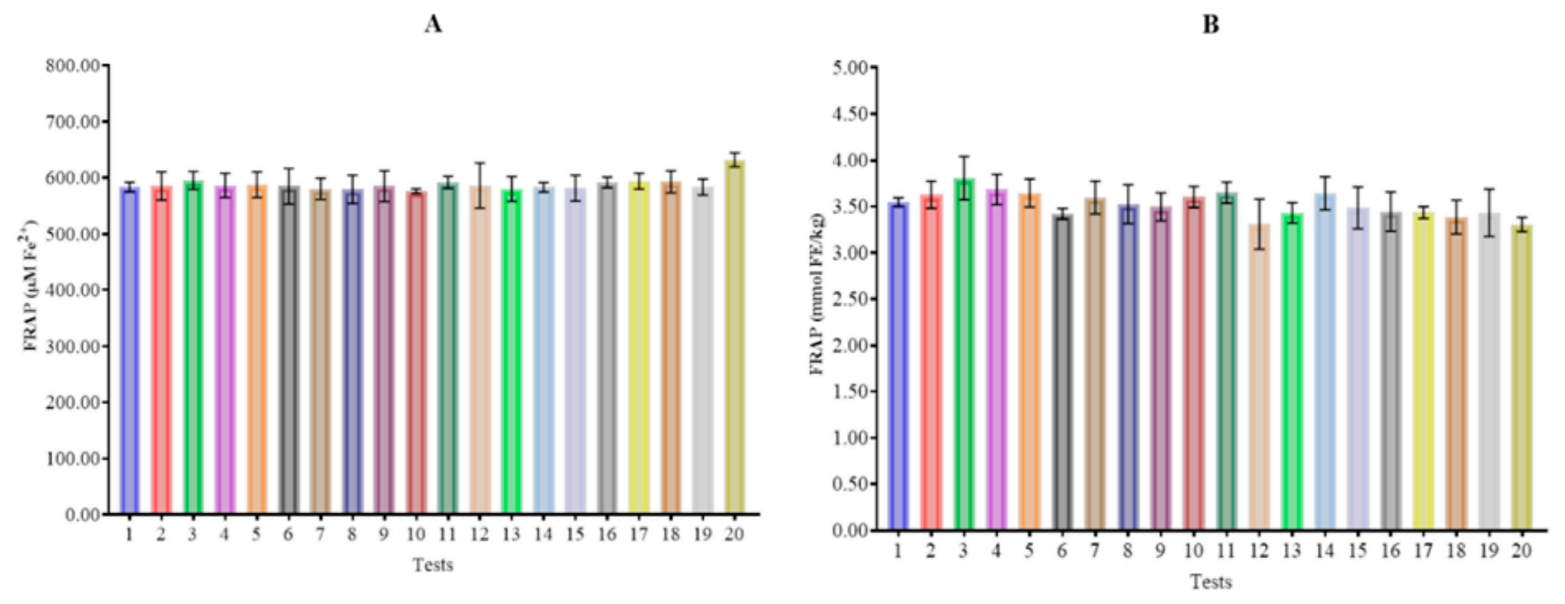
Repeatability of the FRAP assay. Horizontal axis: Day (1–20, non-consecutive testing days). (**A**) 600 µM FeSO_4_·7H_2_O positive control. (**B**) Honey control (Coastal Peppermint). Acceptance criterion for long-term repeatability: RSD ≤ 5.0% (Section 2.7.5).

To evaluate long-term repeatability, two control samples were monitored over 20 non-consecutive testing days: a positive control (600 µM FeSO_4_·7H_2_O) (Figure 8A) and a representative honey sample (Coastal Peppermint honey) (Figure 8B), each analysed in triplicate per day (*n* = 3). The mean FRAP values for the positive control showed minimal variability across the 20 days, indicating stable reagent performance and instrumental response. One-way ANOVA revealed no statistically significant differences across days for either the positive control (*p* = 0.5274) or the honey control (*p* = 0.0505), with all day-to-day variations remaining within predefined analytical acceptance limits.

Importantly, the stability observed for the honey control confirms that the matrix-matched blanking strategy and optimised incubation window effectively suppress matrix-driven drift, allowing the FRAP assay to function as a true quantitative method rather than a relative screening tool for reducing capacity in honey.

### 3.4. Application of the Optimised and Validated FRAP Assay in Honey Analysis

The optimised and validated FRAP assay was applied to 47 Western Australian honey samples representing monofloral (Blackbutt, Jarrah, Manuka, Marri, and Red Bell) and multifloral botanical origins (Table 6). For honey types represented by multiple samples, antioxidant activity is reported as the observed range within each botanical class. This reflects natural compositional variability and is not intended to support formal statistical comparison between floral groups. In addition, the samples were also evaluated using the conventional water-blanked FRAP calculation, to illustrate the extent to which antioxidant activity may be overestimated when matrix contributions are not accounted for.

Among monofloral honeys, Blackbutt and Jarrah exhibited the highest overall antioxidant activities, with honey-blanked FRAP values commonly clustering around approximately 6.4–6.5 mmol Fe^2+^/kg. Marri and Red Bell honeys showed intermediate activities, while Manuka and multifloral honeys generally displayed lower average FRAP values. Substantial within-group variability was observed for all botanical classes. This is consistent with the known influence of floral source, environmental conditions, and nectar chemistry on honey phenolic composition [197,198].

The extent of matrix-related correction was strongly honey-type dependent, with approximately 38% of samples showing statistically significant differences (*p* < 0.05) between water-blanked and matrix-matched results. In several Marri and multifloral samples, blanking corrections exceeded 30% (Table 6). Such large corrections indicate substantial non-reductive contributions from the honey matrix, arising from its inherent colour and/or chelating interactions with Fe^3+^.

The FRAP activities obtained for the Manuka honeys in this study ranged from 2.80 ± 0.09 to 6.44 ± 0.15 mmol Fe^2+^/kg using the matrix-matched honey blank. These values are broadly comparable to those reported using microplate-based FRAP methods by Bolanos de la Torre et al. [187], who observed activities ranging from 2.00 ± 0.61 to 7.60 ± 0.81 mmol Fe^2+^/kg in Manuka honeys with UMF ratings of 5–18 using a 30 min incubation period. Similarly, Habib et al. [188] reported a FRAP activity of approximately 6.0 mmol Fe^2+^/kg for Manuka honey using a comparable incubation time. Direct numerical comparison should nevertheless be interpreted cautiously because the cited studies used different assay configurations and blanking approaches.

Beyond Manuka honey, Ahmad et al. [189] reported that Malaysian Tualang honey exhibited a FRAP activity of 2.56 ± 0.60 mmol Fe^2+^/kg; Habib et al. [188] observed FRAP activities ranging from 5.0 to 12.0 mmol Fe^2+^/kg for honeys from arid and semi-arid regions; and Gašić et al. [199] reported 2.43 ± 0.16 to 30.89 ± 0.68 mmol Fe^2+^/kg across Serbian multifloral and honeydew honeys. These values are broadly comparable to the water-blanked FRAP results obtained in the present study (~2.90 to 10.86 mmol Fe^2+^/kg). The reducing capacities measured here using conventional blanking are therefore consistent with those reported in the literature.

However, most previously published FRAP studies of honey rely on conventional blanking procedures, which do not account for matrix-derived absorbance. This point deserves particular emphasis. Direct numerical comparison with values obtained using the matrix-matched blanking strategy developed here should therefore be interpreted with caution, because methodological differences may influence the reported antioxidant activities. Table 6 demonstrates why: water-blanked values were consistently higher than matrix-matched values across samples. Omitting honey-specific matrix-matched blanking can therefore significantly overestimate reducing capacity in compositionally complex honeys, through the combined effect of non-reductive Fe^3+^ complexation and intrinsic sample absorbance. Consequently, the literature FRAP values obtained by conventional water blanking should be treated as approximate benchmarks rather than directly comparable quantitative metrics against the matrix-matched values reported here.

More broadly, variability among published FRAP values is also influenced by methodological differences. These include reagent composition, incubation time, temperature, detection wavelength, and sample preparation [37,46]. The absence of a universally standardized FRAP protocol further complicates direct inter-study comparison. The present work addresses this limitation by introducing matrix-matched blanking alongside controlled reaction conditions, thereby improving analytical specificity and robustness for honey analysis.

Overall, application of the optimised assay to a range of Western Australian honeys demonstrates that honeys from this region span a wide range of reducing capacities, comparable to those reported globally. It also reveals that the magnitude of matrix-driven bias is strongly dependent on botanical origin. These findings highlight the necessity of honey-specific blanking strategies when the FRAP assay is used to accurately quantify antioxidants in complex natural matrices.

## 4. Conclusions

This study established a validated, high-throughput microplate FRAP protocol specifically optimised for honey through the incorporation of matrix-matched (interaction-corrected) blanking, distinguishing true ferric reduction from non-reductive Fe^3+^–matrix interactions and confirming that sugars do not contribute meaningfully to the FRAP signal. A 30 min incubation at 37 °C in the dark, using 20 µL sample and 180 µL FRAP reagent with absorbance at 620 nm, provided the best compromise between reaction completeness and analytical stability. Validation confirmed excellent performance: linearity for FeSO_4_·7H_2_O over 200–1200 µM, an LOD of 0.28 mmol Fe^2+^ equivalents kg^−1^, an LOQ of 0.94 mmol Fe^2+^ equivalents kg^−1^, precision ≤ 5% RSD, and high repeatability.

Systematic evaluation of representative phenolic compounds revealed that flavonoids exhibit substantially stronger Fe^3+^ interactions than phenolic acids, resulting in large, time-dependent increases in absorbance even in the absence of TPTZ. Epicatechin, naringenin, and myricetin showed the greatest effects, whereas genistin exhibited minimal interaction. Phenolic acids displayed weaker and more heterogeneous behaviour, with gallic and m-coumaric acids showing small net decreases. These findings demonstrate that FRAP responses in phenolic-rich matrices represent a composite of redox activity and metal–ligand interactions unless matrix-matched blanking is applied.

Application of the optimised assay to 47 Western Australian honeys yielded FRAP activities ranging from 2.80 to 9.52 mmol Fe^2+^/kg (mean 5.48 mmol Fe^2+^/kg), providing a standardized reference set for regional honey antioxidant capacity. The matrix-matched blank confers its principal benefit through enhanced assay specificity: removal of non-reductive background readings ensures that reported values reflect only formation of the Fe^2+^–TPTZ chromophore.

A limitation of the present approach is worth noting explicitly: matrix-matched blanking corrects for non-reductive Fe^3+^–matrix interactions, but it does not address the separate issue of slowly reacting antioxidants that may still be under-represented within the 30 min reaction window; incubation time and matrix-matched blanking are complementary but distinct corrections. Future work could extend this framework to other phenolic-rich foods (e.g., wine, tea, and fruit extracts) where iron–polyphenol interactions are similarly expected to confound conventional antioxidant assays and could compare matrix-matched FRAP against DPPH/ABTS measurements made under equivalent matrix-correction principles.

Collectively, this work provides the first systematic experimental characterisation of honey-related Fe^3+^–phenolic interactions within the FRAP assay and demonstrates that matrix-matched blanking is essential for chemically faithful antioxidant measurement. The optimised microplate FRAP protocol offers a robust, reproducible, and analytically specific high-throughput tool for honey research and quality assessment, with potential applicability to other phenolic-rich foods where metal–ligand interactions may confound conventional antioxidant assays.

## Figures and Tables

**Table 1:** Honey varieties used in the study and their botanical origins.

Honey Type	Code	Species	Family	*n*
Blackbutt	BLA	*Eucalyptus patens*	Myrtaceae	6
Jarrah	JAR	*Eucalyptus marginata*	Myrtaceae	13
Manuka	MAN	*Leptospermum scoparium*	Myrtaceae	7
Marri	MAR	*Corymbia calophylla*	Myrtaceae	5
Multifloral	MUL	Various	—	13
Coastal Peppermint *	PEP	*Agonis flexuosa*	Myrtaceae	5
Red Bell	RED	*Calothamnus* spp.	Myrtaceae	3

* used as honey positive control. *n* = number of individually sourced honey samples per botanical type (biological replicates); each was analysed in analytical triplicate (see Section 2.6).

**Table 2:** Condensed summary of FRAP assay parameters reported across 136 honey studies (full study-by-study data in Supplementary Table S1).

Parameter	Key Trend
Detection format	Only 9% of studies used a microplate format; 91% used conventional cuvette-based UV–Vis spectrophotometry [3,5,52,65,66,67,68,69,70,71,72,73]
Sample preparation	53% analysed aqueous honey solutions (35% of all studies at 10% w/v, the single most common concentration); only 4% used solvent extracts [74,75,76,77,78,79]
Incubation time	Ranged 4–90 min; 37% used the original 4 min protocol [37]; 14% used extended 30–90 min incubations
Incubation temperature	74% incubated at 37 °C [80,81,82,83]; 14% at room temperature [72,84,85,86]; only 4 studies (3%) incubated in the dark [73,87,88,89]
Blanking strategy	51% did not specify a blank; among all 136 studies, 22% used water-based blanks, 12% used FRAP reagent alone, and 11% used sugar-analogue (“artificial honey”) matrix blanks
Detection wavelength	Ranged 539–600 nm; 82% used the conventional 593 nm
Result expression	65% ferrous equivalents (FE); 28% Trolox equivalents (TE); 4% ascorbic acid equivalents (AAE)
Normalisation	84% reported results per g, 100 g, or kg of honey

**Table 3:** Ferric-Reducing Antioxidant Power (FRAP) of 20% (*w/v*) artificial honey and sugar solutions.

Sugar	Amount in Floral Honey (%)	µM FeSO_4_ (Mean ± SD)	Remarks	mmol Fe/kg
Artificial honey	N.A.	117.52 ± 6.07	<LOQ	N.A.
Glucose	<30.00 [48]	112.68 ± 5.71	<LOQ	N.A.
Fructose	<50.00 [48]	118.73 ± 4.02	<LOQ	N.A.
Sucrose	<5.00 [191]	145.03 ± 8.86	<LOQ	N.A.
Maltose	<3.60 [192]	95.05 ± 4.27	<LOQ	N.A.
Xylose	<0.50 [193]	117.39 ± 15.85	<LOQ	N.A.
Galactose	<0.02 [194]	149.1 ± 6.24	<LOQ	N.A.
Lactose	<0.04 [194]	115.58 ± 2.75	<LOQ	N.A.
Maltodextrin	N.A.	217.69 ± 12.74	—	1.09 ± 0.06
Mannose	N.A.	91.4 ± 2.11	<LOQ	N.A.

<LOQ = below the limit of quantification; N.A. = not applicable.

**Table 4:** Representative compounds from the full phenolic interaction panel (full 28-compound dataset in Supplementary Table S2).

Compound (Class)	ΔABS 0 → 30 min (%Δ)	FRAP Matrix-Blanked (mmol Fe/kg)	FRAP Water-Blanked (mmol Fe/kg)	% Difference	Category
Quercetin (flavonol)	+1311%	6992.47 ± 15.90	8311.91 ± 21.25	+18.87%	Major Fe^3+^ interaction
Catechin (flavan-3-ol)	+1173%	4470.72 ± 12.93	5295.37 ± 25.44	+18.45%	Major Fe^3+^ interaction
Gallic acid (HBAD)	−34%	7342.65 ± 97.23	7120.35 ± 98.54	−3.03%	No meaningful Fe^3+^ interaction

HBAD = hydroxybenzoic acid and derivatives.

**Table 5:** Accuracy and precision of the FRAP assay (*n* = 3).

Standard	Theoretical Conc. (µM)	Intra-Day Precision (% RSD)	Inter-Day Precision (% RSD)	Accuracy (% Recovery)
FeSO_4_·7H_2_O	960	2.23	2.82	101.24
FeSO_4_·7H_2_O	1200	1.98	1.50	102.70
FeSO_4_·7H_2_O	1440	2.90	2.30	100.75

**Table 6:** Summary of FRAP activity by honey type, matrix-matched vs. water blanking (individual sample data for all 47 honeys in Supplementary Table S3).

Honey Type	*n*	FRAP Matrix-Matched (mmol Fe/kg), Mean (Range)	% Difference vs. Water Blank (Range)	Samples with Significant Difference (*p* < 0.05)
Blackbutt	6	6.46 (4.21–9.42)	2.52–30.08%	2/6
Jarrah	13	6.48 (4.30–8.42)	1.83–28.98%	5/13
Manuka	7	4.93 (2.80–6.44)	2.17–7.86%	2/7
Marri	5	5.23 (3.96–6.57)	2.13–33.33%	3/5
Multifloral	13	4.28 (2.81–9.52)	1.49–32.74%	6/13
Red Bell	3	6.12 (5.54–7.05)	1.04–3.69%	0/3
Overall	47	5.48 (2.80–9.52)	1.04–33.33%	18/47 (38%)

## Data Availability

All data generated or analysed during this study are included in this article and its Supplementary Materials. Further inquiries may be directed to the corresponding author.

## Supplementary Materials

The following supporting information can be downloaded at https://www.mdpi.com/article/10.3390/mps9040120/s1, Table S1: Overview of FRAP Assay Parameters and Antioxidant Activity Measurements in Honey (136 studies); Table S2: Absorbance at 0 and 30 Min and FRAP Activity of Compounds Blanked with Water or Matrix-Matched Blank (28 compounds); Table S3: Application of FRAP Assay in Analysing Western Australian Honeys and Comparison of Antioxidant Activity with and Without Honey Blanking (n = 3 per sample). FRAP Activity Expressed as mmol Fe/kg (Mean ± SD); Figure S1: Chemical Structures of Phenolic Compounds and Related Antioxidants Tested in the FRAP Assay.
