# Supplementary material for: Optimisation and Validation of a Microscale FRAP Assay with Matrix-Matched (Interaction-Corrected) Blanking for Accurate Antioxidant Capacity Assessment of Honeys
Source: Methods Protoc. 2026 Aug 18;9(4):120. doi: 10.3390/mps9040120 (PMC13515261; doi:10.3390/mps9040120)
Supplement: Supplementary file 1 [file mps-09-00120-s001.zip › mps-4372066-supplementary.pdf]

Supplementary Materials

Optimization and Validation of a Microscale FRAP Assay with Matrix-Matched (Interaction-Corrected) Blanking for Accurate Antioxidant Capacity Assessment of Honey

Ivan Lozada Lawag, Sharmin Sultana, Lee Yong Lim, and Cornelia Locher

Contents

Table S1. Overview of FRAP Assay Parameters and Antioxidant Activity Measurements in Honey (136 studies).

Table S2. Absorbance at 0 and 30 Minutes and FRAP Activity of Compounds Blanked with Water or Matrix-Matched Blank (28 compounds)

Table S3. Application of FRAP Assay in Analysing Western Australian Honeys and Comparison of Antioxidant Activity with and Without Honey Blanking (*n* = 3 per sample). FRAP Activity Expressed as mmol Fe/kg (Mean ± SD).

Figure S1. Chemical Structures of Various Phenolic Compounds and Related Antioxidants Tested in the FRAP Assay

Table S1. Overview of FRAP Assay Parameters and Antioxidant Activity Measurements in Honey (136 studies)

Reproduced at full resolution from the submitted manuscript; corresponds to condensed Table 1 in the main text.

Author, Year	FRAP Reagent Vol (µL)	Honey Sample Vol (µL)	Honey Sample Conc (%)	Inc. Time (min)	Inc. Temp. (°C)	Det (nm)	Blank Used	Standard Used	Result Expression	Range of Result	Honey Sample
Wu et al., 2025 [2]	1000	80	10	10	37	593	FR	Trolox	µmol TE/kg	1503.16 ± 61.30 to 1036.92 ± 132.81	Multifloral (China)
Mduda et al., 2025 [64]	4500	500	NI	10	37	593	DW	$FeSO_4•7H_2O$	µmol FE/100g	62.6 to 120.3	Multifloral (Tanzania)
Morar et al., 2025 [65]	190	10	10	30	RT	593	FR	Trolox	µg TE/g	121.20 ± 11.27 to 148.85 ± 14.09	Manuka, Ohia Lehua (Romania)
Qi et al.2025 [68]	200	6	50	10	RT	593	UW	$FeSO_4•7H_2O$	mmol FE/kg	7.71 ± 1.46	C. pi. Shen (China)
Mohammadi et al., 2024 [90]	3000	100	20	4	37	593	FR	Trolox	µM TE/100g	67 to 606	Eucalyptus, Annaab, Jangale, Chand Giah, Sumaq, Gaz (Iran)
Alarjani et al., 2024 [91]	1500	200	20	4	37	593	DW	$FeSO_4•7H_2O$	mg Fe^{3+}E/100g	142.20 ± 83.16 to	Acacia Asir, Acacia Jazan, Ziziphus Asir,

										580.20 ± 112.28	Ziziphus Jazan, Asir, Multifloral Jazan (Saudi Arabia)
Kolayli et al., 2024 [92]	3000	100	NI	4	37	595	NI	$FeSO_4•7H_2O$	µmol FE/100g	124.20 ± 4.01 to 248.70 ± 6.65	Lavender (Türkiye)
Hajian-Tilaki et al., 2024 [93]	1800	200	10	10	37	593	DW	$FeSO_4•7H_2O$	µM FE/100g	80.20 to 154.65	Multifloral (Iran)
Fernandes et al., 2024 [3]	180	20	20	30	37	594	NI	$FeSO_4•7H_2O$	µmol FE/kg	654 to 5466	Leptospermum, Banksia, Blueweed/Lucerne (Australia)
Mduda et al., 2024 [94]	4500	500	NI	10	37	595	NI	$FeSO_4•7H_2O$	µmol FE/100g	53.50 to 187.78	Multifloral (Tanzania)
Karahalil et al., 2024 [95]	1500	50	NI	4	37	595	NI	Trolox	µmol TE/g	0.47 to 0.75	Chestnut, Mad (Rhododendron) (Türkiye)
Harbane et al., 2024 [96]	2700	300	5	6	37	593	NI	Trolox	µmol TE/g	50.7 to 237.2	*Ceratonia siliqua*, Eucalyptus, Arbutus, Honeydew (Algeria)
Mduda et al. 2023 [97]	4500	500	NI	10	37	595	NI	$FeSO_4•7H_2O$	µmol FE/100g	68.11 to 221.23	Acacia (Tanzania)

Ismail et al. 2023 [98]	NI	NI	NI	NI	NI	NI	NI	Trolox	µmol TE/100g	249.31 to 716.23	Ziziphus, Acacia (Sudan)
Kemal et al. 2023 [99]	3000	100	NI	4	37	595	NI	$FeSO_4 \bullet 7H_2O$	µmol FE/100g	143.00 to 214.00	Black Cumin (Türkiye)
Uçar et al. 2023 [100]	3000	100	NI	4	37	595	NI	$FeSO_4 \bullet 7H_2O$	µmol FE/100g	126.9 to 239.1	Multifloral (Northern Cyprus)
Saral et al. 2023 [101]	3000	100	NI	4	37	595	NI	$FeSO_4 \bullet 7H_2O$	µmol FE/100g	175.61 to 432.03	Chestnut (Türkiye)
Kolayli et al. 2023 [4]	3000	100	NI	4	37	595	NI	$FeSO_4 \bullet 7H_2O$	µmol FE/100g	219.50 ± 8.67	Black Cumin (Türkiye)
Fernández-Estellé et al., 2023 [102]	600	100	NI	5	NI	595	NI	Trolox	mg TE/g	0.2 to 2.2	Eucalyptus, Orange/Lemon, Thyme, Rosemary, Heather, Forest, Holm Oak, Mountain (Spain)
Dong et al., 2023 [66]	180	20	20	30	37	594	NI	$FeSO_4 \bullet 7H_2O$	µmol FE/kg	3,268 to 4,468	Jarrah, Manuka (Australia)
Mohd Shahabuddin et al., 2022 [67]	190	10	NI	60	NI	594	NI	$FeSO_4 \bullet 7H_2O$	mM	0.439 to 0.566	Kelulut – Multifloral (Malaysia)
Sahin et al., 2022 [103]	3000	100	NI	4	37	595	NI	$FeSO_4 \bullet 7H_2O$	µmol FE/g	2.905 to 4.415	Pine (Türkiye)

Puścion-Jakubik et al., 2022 [69]	180	20	NI	10	37	593	NI	$FeSO_4 \cdot 7H_2O$	µM FE/mL	0.014 to 0.394	Chestnut, Eucalyptus, Lemon, Orange, Acacia, Buckwheat, Dandelion, Heather, Honeydew (Coniferous), Honeydew (Deciduous), Linden, Phacelia, Rape, Raspberry, Almond, Heath, Lavender, Rosemary, Thyme (Poland)
Qahtani et al., 2022 [70]	180	20	NI	10	37	593	NI	Trolox	mg TE/g	7.40 to 32.32	Sidr, Talh (Saudi Arabia)
Jurič et al., 2022 [104]	1800	200	5	10	37	593	SA	$FeSO_4 \cdot 7H_2O$	mmol FE/kg	13.5	Strawberry Tree (Croatia)
Wu et al., 2022 [105]	1000	80	10	10	37	593	NI	Trolox	µmol TE/kg	1768.10 to 5217.06	Multifloral (China)
Otmani et al., 2021 [85]	NI	500	2.5	5	RT	593	NI	Gallic Acid	mg GAE/100 g	9.54 to 257.22	Monofloral, Multifloral (Algeria)

Hunter et al., 2022 [53]	1800	100	NI	NI	37	593	DW, Saliva	Trolox	mmol TE/g	0.670 to 5.53	Manuka, Eucalyptus, Leatherwood, Clover, Red Gum, Yellow Box, Stringybark (Australia)
Balkanska and Shumkova, 2022 [106]	3000	100	NI	NI	37	593	NI	Fe^{2+}	µM Fe^{2+}	0.42 to 1.14	Multifloral, Sunflower (Bulgaria)
Shamsudin et al., 2022 [74]	1500	200	0.05	10	37	593	NI	$FeSO_4•7H_2O$	µmol FE/100 g	72.78 to 164.88	Acacia, Gelam, Starfruit (Malaysia)
Bouacha et al., 2022 [107]	1800	200	1	10	37	593	NI	Ascorbic acid	µmol AAE/g	231.85 ± 1.78 to 1271.42 ± 2.13	Eucalyptus, Pinus, Quercus, Castanea, Citrus, Thyme, Rue, Rosemary, Pituranthos, Marrubium, Ecballium, Lonicera, Ziziphus, Artemisia (Algeria)
Moein et al., 2022 [71]	180	20	NI	10	37	593	NI	$FeSO_4•7H_2O$	IC_{50} (mg/mL)	0.0018 to >3.200	Gavan, Zataria, Bahareh, Meymand (Iran)

Lazarus et al., 2021 [108]	1800	200	10	10	37	593	SA	FeSO4	μmol FE/kg	72.2 to 510	Chestnut, Black Locust, Honeydew, Sage, Buckthorn, Savory, Multifloral (Croatia)
Ng et al., 2021 [109]	NI	NI	20	4	37	593	NI	$FeSO_4$•$7H_2O$	mmol FE/kg	1.33 to 3.58	Multifloral
Olate-Olave et al., 2021 [110]	1500	50	30	15	RT	593	NI	$FeSO_4$•$7H_2O$	mmol Fe (II) E/100g	0.7 to 1.6	Multifloral (Chile)
Alkan et al., 2020 [84]	NI	NI	NI	NI	RT	595	NI	Trolox	mM TE/g	0.409 to 6.177	Rhododendron (Turkey)
Mat Ramlan et al., 2021 [72]	220	20	NI	NI	RT	593	M	$FeSO_4$•$7H_2O$	μg FE/g	0.1 to 4000	Multifloral (Malaysia)
Ganaie et al., 2021 [111]	NI	NI	NI	4	RT	593	NI	Trolox	μmol FE/g	0.72 to 1.49	Black Locust, Multifloral, Plectranthus (India)
Kulkarni et al., 2021 [87]	1800	200	10	10	RT	593	SA	$FeSO_4$•$7H_2O$	μM FE/100 g	760 to 1050	Ajwain (India)
Ara et al., 2021 [112]	1500	50	NI	NI	NI	593	HS+AB	Ascorbic Acid	μM AAE/mg	319.77 ± 9.4	Multifloral (Pakistan)

Kędzierska-Matysek et al., 2021 [113]	1800	200	10	10	37	593	DW	Trolox	mM TE/kg	0.29 to 2.14	Rapeseed, Buckwheat, Linden, Black Locust, Multifloral (Poland)
Cacique et al., 2021 [114]	3000	100	NI	45	37	593	NI	Trolox	mg TE/g or L	0.1743 to 0.3645	Multifloral (Brazil)
Ooi et al.2021 [5]	175	25	10	4	37	593	NI	Ascorbic Acid	µM AAE/100 g	206.77 ± 1.76	Multifloral (Malaysia)
Majkut et al., 2021 [115]	2000	200	NI	10	37	593	NI	Trolox	mmol TE/100 g	0.083 to 0.95	Multifloral, Lime, Rapeseed, Buckwheat (Poland)
Chen et al., 2021 [116]	1500	200	10	4	37	593	NI	$FeSO_4{\bullet}7H_2O$	µmol FE/100 g	179.71 to 344.20	Multifloral (Malaysia)
Laaroussi et al., 2021 [52]	290	10	20	15	37	593	NI	$FeSO_4{\bullet}7H_2O$	mmol FE/g	0.025 ± 0.00	Multifloral (Morocco)
Starowicz et al., 2021 [82]	900	150	NI	5	37	593	NI	Trolox	mmol TE/g	5.29 to 47.13	Acacia, Buckwheat, Heather, Linden, Multifloral, Rapeseed (Poland)
Mahmood et al., 2021 [117]	1500	200	NI	4	37	593	DW	$FeSO_4{\bullet}7H_2O$	µg Fe^{2+}/Kg	42.31 to 153.26	Multifloral (Malaysia)

Zhu et al., 2020 [75]	NI	500	0.20	NI	NI	593	NI	Trolox	mg TE/kg	2.04 ± 0.29	*Amorpha fruticosa* (China)
Pentoś et al., 2020 [118]	3000	1000	NI	10	NI	593	NI	Trolox	µM TEAC/100g	417.36 to 2076.63	Multifloralr, Heather, Buckwheat, Honeydew, Manuka (Poland)
Degirmenci et al., 2020 [119]	3000	100	NI	4	37	593	DW	Trolox	µmol TE/g	0.324 to 1.434	Multifloral (Azerbaijan)
Ibrahimi and Hajdari, 2020 [120]	3000	100	NI	4	NI	593	FR	Trolox	mg TE/100g	3.65 to 22.39	Meadow, Acacia, Forest, Mixed, Chestnut, Pine, Lime (Kosovo)
Missio da Silva et al. 2020 [121]	1800	200	NI	10	37	594	FR	$FeSO_4 \bullet 7H_2O$	µmol Fe (II)E/100g	70 to 350	Multifloral, *Baccharis leucocephala*, *Hovenia dulcis* (Brazil)
Nayaka et al., 2020 [122]	NI	NI	NI	30	25	593	AB	Ascorbic Acid	AAI	0.182 to 4874	Multifloral (Indonesia)
Cianciosi et al., 2020 [54]	1500	150	NI	4	RT	593	NI	$H_8FeN_2O_8S_2 \cdot 6 H_2O$	µmol FE/100 g	4.50 to 261.73	Manuka (Italy)
Wan Yaacob et al., 2020 [88]	1800	200	20,0.05	15	37	593	NI	$FeSO_4 \bullet 7H_2O$	mmol FE/mg	2.90 to 37.56	Tualang – Multifloral (Malaysia)

Erturk et al., 2019 [123]	3000	100	10	4	37	593	NI	Trolox	µmol TE/100 g	1.00 to 5.49	Chestnut, Lavandula, Acacia, Sunflower, Thyme, Clover, Astragalus (Turkey)
Zarei et al., 2019 [83]	2800	200	50	15	37	593	NI	$FeSO_4$•$7H_2O$	µM FE/50 %	50 to 750	Alfalfa, Milkvetch, Lotus, Thyme, Multifloral (Iran)
Zarei et al., 2019 [124]	2800	200	50	15	37	593	NI	$FeSO_4$•$7H_2O$	µM FE/50 %	400 to 800	Lotus, Thyme, Multifloral (Iran)
Tuksitha et al., 2018 [125]	180	6	NI	NI	37	600	NI	FeSO4	mM Fe^{2+}/100 g	25.78 to 50.66	Multifloral (Malaysia)
Perna et al., 2019 [126]	2900	100	10	30	37	593	DW+FR	$FeSO_4$•$7H_2O$	mg TE/100 g	9.87	Sulla (Italy)
Malkoç et al., 2019 [127]	300	100	NI	4	37	595	DW	Trolox	µmol TE/100 g	92.53 to 124.33	Anzer - Multifloral (Türkiye)
Živković et al., 2019 [86]	3000	100	NI	30	RT	593	FR+AB	$FeSO_4$•$7H_2O$	mmol FE/kg	0.83 to 6.04	Acacia, Linden, Oregano, Lavender, Honeydew, Meadow, Forest (Serbia)

Saral et al., 2019 [128]	3000	100	NI	4	RT	593	FR	$FeSO_4 \bullet 7H_2O$	µmol FE/g	1.37 to 4.57	Multifloral (Türkiye)
Turkut et al., 2018 [80]	3000	100	NI	4	37	595	HS+DW	Trolox	µmol TE/g	341 ± 38 to 6134.373 ± 293.0	Chestnut, Pine, Multifloral (Türkiye)
Kolayli et al., 2018 [81]	NI	NI	NI	4	37	593	FR	$FeSO_4 \bullet 7H_2O$	µmol FE/kg	439±5 to 1015±3	Oak (Türkiye)
Chaiyasut et al., 2018 [129]	1800	60	NI	4	RT	539	NI	$FeSO_4 \bullet 7H_2O$	mg FE/g	0.33 to 0.48	Hevea, Sesame, Eupatorium, Sunflower, Rambutan, Longan (Thailand)
Nascimento et al., 2018 [130]	NI	NI	20	NI	37	593	NI	Trolox	µmol TE/g	0.22 to 2.11	Eucalyptus, Japanese grape, Mastic, Quitoco, Wildflower, Multifloral (Brazil)

Moloudian et al., 2018 [131]	1800	200	10	10	37	593	SA	$FeSO_4{\bullet}7H_2O$	µM Fe (II)/10%	56.8 to 582.2	Milk Vetch, Thyme, Christ's Thorn, Jujube, Eucalyptus, Barberry, Manna of Camelthorn, Coriander, Acanthus, Sunflower, Dill, Parsley, Alfalfa, Cotton, Hami Melon, Pear, Honeydew, Persian Manna (Iran)
Kek et al., 2017 [73]	190	10	10	30	NI	593	FR	Ascorbic acid	mg AAE/100 g	19.05 to 41.95	Tualang, Gelam, Pineapple, Multifloral, Kelulut (Malaysia)
Grabek-Lejko et al., 2018 [132]	1800	200	NI	10	RT	593	NI	Trolox	mmol TE/kg	0.79 to 3.73	Rape, Lime, Buckwheat, Multifloral, Honeydew (Poland)
de Oliveira Neto et al., 2017 [133]	1800	200	20	10	37	593	SA	$FeSO_4{\bullet}7H_2O$	µmol FE/g	10.83 to 407.45	Multifloral (Brazil)

Ahmad et al., 2017 [198]	300	5	NI	30	37	593	NI	$FeSO_4 \bullet 7H_2O$	mmol FE/100 g	255.5 ± 6.01	Tualang – Multifloral (Malaysia)
Nweze et al., 2017 [134]	1800	200	10	10	37	593	SA	$FeSO_4 \bullet 7H_2O$	µM Fe (II) E/100 g	426.93 to 666.88	Multifloral (Nigeria)
Zhao et al., 2017 [76]	NI	500	0.02	NI	37	593	NI	Trolox	mg TE/kg	191.8 to 317.4	Multifloral (China)
Petretto et al., 2017 [135]	NI	NI	20	NI	NI	593	NI	$FeSO_4 \bullet 7H_2O$	mmol FE/kg	0.96 to 4.74	Thyme, Citrus, Carob, Euphorb, Eucalyptus, Orange, Multifloral (Morocco)
Kuś et al., 2017 [136]	2000	20000	20	NI	NI	593	NI	$FeSO_4 \bullet 7H_2O$	mmol FE/kg	3.2 ± 1.1	Fir Honeydew (Poland)
Soares et al., 2017 [137]	2700	90	NI	15	37	595	NI	$FeSO_4 \bullet 7H_2O$	mg FE/kg	1391.2 to 5130.2	Heather, Lavender, Linden, Almond, Blueberry, Chestnut, Orchard, Multifloral (Portugal)
Sowa et al., 2017 [138]	NI	NI	20	4	37	593	NI	Trolox	µmol TE/kg	1033.33 to 2779.17	*Melilotus albus* (Poland)

Afrin et al., 2017 [139]	1800	200	NI	10	37	593	NI	Trolox, Fe (II)	mmol TE/100 g, mmol Fe^{2+} /100 g	0.14 ± 0.00 to 0.54 ± 0.00, 0.29 ± 0.00 to 0.92 ± 0.02	Strawberry Tree (Italy)
Jerković et al., 2017 [140]	2000	20	20	NI	NI	593	NI	$FeSO_4$•$7H_2O$	mmol FE/kg	5.76 ± 0.66	*Moltkia petraea* (Croatia)
Hagr et al., 2017 [141]	3000	400	10	30	37	593	FR	Trolox	µM TE/100 g	273.33 ± 1.4 to 281.08 ± 1.4	Multifloral (Sudan)
Ahmed et al., 2016 [142]	1500	200	10	4	37	593	HPW	$FeSO_4$•$7H_2O$	µM Fe^{2+}/100 g	238.21 to 672.02	Jujube, Acacia, Multifloral (Pakistan)
Kolayli et al., 2016 [143]	3000	100	NI	4	37	595	DW+FR	Trolox	µM TE/100 g	355 ± 23.08 to 462 ± 18.48	Chestnut (Türkiye)
Sahin 2016 [144]	3000	100	NI	4	RT	593	FR	$FeSO_4$•$7H_2O$	µmol FE/g	0.527 ± 0.013 to 4.690 ± 0.094	Chestnut, Oak, Multifloral (Türkiye)
Shamsi et al., 2014 [6]	1500	200	NI	5	RT	593	FR	Ascorbic Acid	mM Fe^{2+}E/mL	0.078 to 0.628	Multifloral (India)

Bundit et al., 2016 [145]	900	30	10	4	37	593	NI	$FeSO_4•7H_2O$	µM FE/kg	605.55 to 9,083.33	Longan, Sunflower, Lychee, Korlan, Sesame, Rambutan, Para Rubber, Eucalyptus, Coconut, Mangosteen, Macadamia, Bittervine, Manuka (Thailand)
Sahin et al., 2015 [146]	3000	100	10	4	37	595	DW+FR	Trolox	µM TE/g	0.491 to 1.616	Mad *(Rhondendron spp.)* (Türkiye)
Khalil et al., 2015 [147]	1500	200	10	4	37	593	DW	$FeSO_4•7H_2O$	µmol FE/100 g	249.1 to 303.9	Tualang – Multifloral (Malaysia)
Jerković et al., 2015 [148]	2000	20	20	NI	NI	593	NI	$FeSO_4•7H_2O$	mmol FE/kg	2.5 to 5.1	*Satureja montana* L. (Croatia)
Wang et al., 2015 [149]	NI	20	NI	NI	NI	593	NI	Trolox	mg TE/kg	96.69 to 132.36	Vitex (China)
Bolanos de la Torre et al., 2015 [188]	1500	75	10	30	37	593	NI	$FeSO_4•7H_2O$	mmol FE/100 g	0.20 to 0.76	Manuka (UK)

Das et al., 2015 [150]	3000	400	10	4	37	593	HS+DW	$FeSO_4•7H_2O$	µMol FE/L	$2.75 \times 10^6 \pm 4.8$	Sesame (India)
Ng and Lim 2015 [151]	1500	200	10	4	37	593	NI	$FeSO_4•7H_2O$	µM FE/kg	196.0 to 643.3	Melaleuca (Malaysia)
Cheng et al., 2015 [78]	4000	300	0.02	4	37	593	NI	Trolox	mg TE/g	0.355 ± 0.05	Buckwheat (China)
Yap & Mohd Fadzelly 2015 [152]	3000	100	NI	90	37	593	FR	$FeSO_4•7H_2O$	µM F E/g	6.79 ± 0.02 to 46.73 ± 0.06	Multifloral - Wild, Commercial, Mangrove, Upper Mountain, Potiukan, Tropical, Manuka (Malaysia)
Yildiz et al., 2014 [153]	3000	100	NI	4	37	595	DW+FR	Trolox	mmol TE/g	24.11 ± 1.10	Chestnut (Türkiye)
Perna et al., 2014 [55]	2900	100	10	30	37	593	DW+FR	Ascorbic Acid	µg AAE/mL	92.88 to 253.29	Chestnut and Sulla (Italy)
Habib et al., 2014 [197]	2000	1000	NI	30	37	593	DW	$FeSO_4•7H_2O$	µmol Fe (II) E/g	500 to 1300	Ghaf, Wild Jujube, Wild Mountain, Marya Herbal, Multifloral, Herbs, Mountain Herbs, Manuka,

											Black Forest (UAE)
Dor et al., 2014 [154]	2850	150	NI	30	RT	593	FR	Trolox	mmol TE/100 g	160.77 to 170.50	Eucalyptus, Litchi, Longan (Mauritius)
Gambacorta et al., 2014 [155]	NI	200	10	15	37	593	SA	$FeSO_4 \bullet 7H_2O$	µM FE/kg	98.26 to 786.53	Sulla (Italy)
Moniruzzaman et al., 2014 [156]	1500	200	10	4	37	593	DW	$FeSO_4 \bullet 7H_2O$	µM FE/100 g	116.00 to 786.22	Mustard, Kalijira, Padma, Sesame (Bangladesh)
Cheng et al., 2014 [77]	4000	500	0.02	NI	NI	593	DDW	Trolox	mg TE/kg	180.32 to 379.82	Jujube (China)
Ertürk et al., 2014 [157]	3000	100	NI	60	37	593	DW	$FeSO_4 \bullet 7H_2O$	µmol FE/g	0.973 to 9.053	Multifloral (Türkiye)
Kuś et al., 2014 [158]	2000	20	NI	4	37	593	NI	$FeSO_4 \bullet 7H_2O$	mmol FE/kg	0.6 to 5.7	Rapeseed, Heather, Lime, Black Locust, Buckwheat, Goldenrod (Poland)
Kuś et al., 2014 [159]	2	20000	20	NI	NI	593	NI	$FeSO_4 \bullet 7H_2O$	mmol FE/kg	1.5 ± 0.7	Cornflower (Poland)

Gorjanović et al., 2013 [89]	1800	200	10	10	37	593	SA	Trolox	μmol TE/g	0.20 to 4.98	Forest, Pine, Urtica, Meadow, Linden Acacia (Serbia)
Chua et al., 2013 [160]	1800	200	10	10	37	593	NI	Trolox	mg TE/100 g	52.39 to 82.53	Tualang, Gelam, Acacia (Malaysia)
Maksimović and Nedić 2013 [161]	NI	100	10	30	37	593	SA	$FeSO_4 \bullet 7H_2O$	μmol FE/100 g	243.3 to 3089.8	Honeydew, Multifloral (Serbia)
Moniruzzaman et al., 2013 [162]	NI	200	10	4	37	593	DW	$FeSO_4 \bullet 7H_2O$	μM Fe^{2+}E/100 g	193.31 to 379.53	Acacia (Malaysia)
Grabek-Lejko et al., 2013 [163]	2000	200	20	10	RT	593	NI	$FeSO_4 \bullet 7H_2O$	mmol FE/kg	1.32 ± 0.04 to 5.74 ± 0.09	Buckwheat, Rape, Multifloral (Poland)
Jubri et al., 2013 [164]	1200	40	10	4	37	593	SA	$FeSO_4 \bullet 7H_2O$	μM FE/kg	215.71 ± 50.00	Manuka (Malaysia)
Tuberoso et al., 2013 [165]	2000	20	10, 20	4	RT	593	NI	$FeSO_4 \bullet 7H_2O$	mmol FE/kg	12.0 ± 2.2	Strawberry Tree (Italy)
Srimeena et al., 2013 [166]	3000	200	10	4	37	593	NI	FeSO4	μM Fe^{2+}/100 g	15.29 to 79.21	Multifloral (India)
Moniruzzaman et al., 2013 [167]	1500	200	10	4	37	593	DW	$FeSO_4 \bullet 7H_2O$	μM Fe^{2+}/100 g	209.78 to 653.75	Gelam, Longan, Rubber Tree, Sourwood,

											Manuka (Malaysia)
Schneider et al., 2013 [168]	1000	30	NI	4	37	593	DW+FR	$FeSO_4 \bullet 7H_2O$	mM FE/kg	0.19 to 3.08	Multifloral, Manuka (UK)
Šarić et al., 2013 [169]	3600	400	10	10	37	593	NI	$FeSO_4 \bullet 7H_2O$	µM FE/100 g	0 to 410.50	Acacia, Chestnut (Türkiye)
Islam et al., 2012 [170]	1500	200	10	4	37	593	DW	$FeSO_4 \bullet 7H_2O$	µmol FE/100 g	140.23 to 772.4	Black Seed, Sesame, Litchi, Ramtil, Mustard, Multifloral (Bangladesh)
Šarić et al., 2012 [171]	3600	400	10	10	37	593	NI	$FeSO_4 \bullet 7H_2O$	mM FE/10 %	18.83 to 319.41	Acacia, Multifloral (Croatia)
Perna et al., 2012 [172]	1800	200	10	15	37	593	AH	$FeSO_4 \bullet 7H_2O$	µM Fe^{2+}	152.65 to 881.34	Chestnut, Sulla, Citrus, Eucalyptus, Multifloral (Italy)
Khalil et al., 2012 [173]	1500	200	10	4	37	593	NI	$FeSO_4 \bullet 7H_2O$	µM FE/kg	221.41 to 281.38	Tualang, Multifloral from Borneo Tropical Vegetation (Malaysia)

Lianda et al., 2012 [79]	4500	500	0.02	10	37	593	M+FR	$FeSO_4 \bullet 7H_2O$	mmol FE/100 g	34.99 to 438.69	Multifloral, Orange (Brazil)
Sant'Ana et al., 2012 [174]	4500	500	10	10	37	593	M+FR	$FeSO_4 \bullet 7H_2O$	µmol FE/100 g	66.28 to 388.50	Cambara, Morrão de Candeia, Eucalyptus (Brazil)
Khalil et al., 2011 [175]	1500	200	10	4	37	593	DW	$FeSO_4 \bullet 7H_2O$	µmol FE/kg	363.38 to 1295.34	Tualang, Gelam, Borneo Tropical, Manuka (Malaysia)
Kishore et al., 2011 [176]	1500	NI	NI	4	37	593	FR	$FeSO_4.7H_2O$	µmol FE/kg	47.92 to 121.89	Tualang, Gelam, Indian forest, Pineapple (Malaysia)
Tezcan et al., 2011 [177]	3000	100	NI	4	37	593	DW	$FeSO_4 \bullet 7H_2O$	mmol FE/kg	31.32 to 346.24	Chestnut, Rhododendron, Multifloral, Acacia, Pine (Türkiye)
Ulusoy et al., 2010 [178]	3000	100	NI	4	37	593	DW+FR	$FeSO_4 \bullet 7H_2O$	µM FE/mg	33 to 166	Lime, Rhododendron, Chestnut, Anzerr, Mutifloral (Türkiye)
Lachman et al., 2010 [179]	3000	10	0.5	NI	37	593	NI	Ascorbic Acid	mg AAE/kg	295.35 to 776.05	Multifloral, Lime, Raspberry, Rape, Honeydew (Czechia)

Mohamed et al., 2009 [180]	1500	200	10	4	37	593	DW+FR	$FeSO_4•7H_2O$	μM Fe^{2+}E/L	322.1 ± 9.7	Tualang (Malaysia)
Tuberoso et al., 2009 [181]	NI	NI	NI	NI	NI	593	NI	$FeSO_4•7H_2O$	mmol FE/kg	245.8 to 425.3	Asphodel (Italy)
Pichichero et al., 2009 [182]	1800	200	10	60	37	593	SA	$FeSO_4•7H_2O$	mmol FE/kg	1.265 to 4.396	Acacia, Ailanthus, Chestnut, Orange, Savory, Sulla, Thymus, Honeydew, Multifloral (Italy)
Krpan et al., 2009 [183]	1800	200	10	10	37	593	SA	$FeSO_4•7H_2O$	μM FE/10 %	6.95 to 142.43	Acacia (Croatia)
Bertoncelj et al., 2007 [184]	1800	200	10	10	37	593	SA	$FeSO_4•7H_2O$	μM FE/10 %	71.0 to 478.5	Acacia, Lime, Chestnut, Fir, Spruce, Multifloral, Forest, Honeydew (Latifoliae and Coniferous) (Slovenia)
Blasa et al., 2006 [185]	1800	200	10	10	37	593	FR	$FeSO_4•7H_2O$	μM FE/10 %	61.75 to 124.5	Millefiori, Acacia (Italy)

Beretta et al., 2005 [28]	NI	50	10	NI	37	593	SA	$FeSO_4 \bullet 7H_2O$	μM FE/10 %	79.5 to 1501.4	Strawberry Tree, Buckwheat, Chestnut, Sulla, Clover, Dandelion, Chicory, Acacia, Mountain Multifloral, Honeydew (Italy)
Aljadi and Kamaruddin, 2004 [186]	300	10	10	4	37	593	FR	$FeSO_4 \bullet 7H_2O$	μmol Fe^{2+}/kg	961 to 1345	Gelam, Coconut (Malaysia)

Legend: Inc – incubation; Det – detection; FR – FRAP reagent; DW – distilled water; DDW – double-distilled water, UW – ultrapure water; HPW – high-purity water; NI – not indicated (no information provided); SA – sugar analog; AH –artificial honey; DW, saliva – distilled water with saliva; M – methanol; HS+AB – honey sample with acetate buffer; AB – acetate Buffer; DW+FR – distilled water with FRAP reagent; FR+AB – FRAP reagent with acetate buffer; HS+DW – honey sample with distilled water; AAE – ascorbic acid equivalent; FE – ferrous ion equivalent; TE – trolox equivalent; GAE – gallic acid equivalent; IC50 – concentration required to achieve 50% inhibition; AAI – antioxidant activity index

Table S2. Absorbance at 0 and 30 Minutes and FRAP Activity of Compounds Blanked with Water or Matrix-Matched Blank (28 compounds)

Compound (Class, Code)	CAS Number	ABS (620 nm, Matrix-Matched Blank, 0 min) (Mean ± SD)	ABS of Matrix-Matched Blank at 30 min (Mean ± SD)	Δ ABS of Matrix-Matched Blank at 0 min vs 30 min ± SD (Percent Change ± SD)	*p*-value (Absorbance Change: 30 min vs. 0 min, Matrix-Matched Blank)	FRAP Activity (mmol Fe/kg, Matrix - Matched Blanked Interaction))	FRAP Activity (mmol Fe/kg, Water-Matrix-Matched Blanked – Without Compound Interaction Correction)	Δ FRAP Activity (mmol Fe/kg) ± SD (Effect of Sample vs. Water Blanking Correction)	p-value (FRAP Activity Difference: Sample-Blanked vs. Water-Blanked Correction)	Category (Fe^{3+} Interaction & Potential FRAP Interference)
Water (Control)	7732-18-5	0.073 ± 0.014	0.088 ± 0.011	0.016 ± 0.017 (21.56 ± 4.84 %)	1.63×10^{-2} (MS)	NA	NA	NA	NA	No meaningful Fe^{3+} interference
Apigenin, a flavone (1)	520-36-5	0.027 ± 0.005	0.394 ± 0.005	0.368 ± 0.008 (1378.75 ± 30.08 %)	1.60×10^{-4} (S)	342.69 ± 18.40	1439.83 ± 29.35	1097.14 ± 17.69 (320.16 ± 16.36 %)	8.66×10^{-5} (HS)	Major Fe^{3+} interaction
Chrysin, a flavone (2)	480-40-0	0.027 ± 0.001	0.334 ± 0.006	0.307 ± 0.007 (1137.04 ± 26.38 %)	1.84×10^{-4} (S)	812.38 ± 8.28	1694.40 ± 28.69	882.02 ± 21.91 (108.57 ± 1.99 %)	2.06×10^{-4} (S)	Major Fe^{3+} interaction

Luteolin, a flavone (3)	491-70-3	0.057 ± 0.005	0.414 ± 0.005	0.357 ± 0.001 (626.90 ± 1.01%)	8.70 × 10^{-7} (HS)	5037.22 ± 14.35	6206.07 ± 24.41	1168.85 ± 16.94 (23.20 ± 0.33 %)	7.00 × 10^{-5} (HS)	Strong Fe^{3+} interaction
Fisetin, a flavonol (4)	528-48-3	0.054 ± 0.002	0.503 ± 0.004	0.448 ± 0.006 (825.15 ± 10.14%)	5.03 × 10^{-5} (HS)	5803.30 ± 9.02	7288.87 ± 14.93	1485.57 ± 14.49 (25.60 ± 0.26 %)	3.17 × 10^{-5} (HS)	Major Fe^{3+} interaction
Myricetin, a flavonol (5)	529-44-2	0.046 ± 0.003	0.690 ± 0.003	0.645 ± 0.006 (1411.68 ± 13.91%)	3.23 × 10^{-5} (HS)	6676.95 ± 18.40	8835.39 ± 17.69	2158.43 ± 11.53 (32.33 ± 0.22 %)	9.50 × 10^{-6} (HS)	Major Fe^{3+} interaction
Quercetin, a flavonol (6)	117-39-5	0.032 ± 0.003	0.456 ± 0.003	0.424 ± 0.001 (1311.34 ± 3.09%)	1.85 × 10^{-6} (HS)	6992.47 ± 15.90	8311.91 ± 21.25	1319.44 ± 16.35 (18.87 ± 0.11 %)	2.05 × 10^{-5} (HS)	Major Fe^{3+} interaction
Hesperidin, a flavanone (7)	520-26-3	0.023 ± 0.001	0.284 ± 0.001	0.260 ± 0.001 (1115.71 ± 2.47%)	1.64 × 10^{-6} (HS)	371.04 ± 18.27	1071.72 ± 19.10	700.68 ± 12.12 (188.98 ± 9.55 %)	2.04 × 10^{-5} (HS)	Strong Fe^{3+} interaction
Naringenin, a flavanone (8)	67604-48-2	0.023 ± 0.001	0.360 ± 0.003	0.337 ± 0.004 (1488.24 ± 17.83%)	4.78 × 10^{-5} (HS)	236.32 ± 24.65	1210.36 ± 19.89	974.04 ± 18.67 (415.56 ± 47.31 %)	4.06 × 10^{-5} (HS)	Major Fe^{3+} interaction
Naringin, a flavanone (9)	10236-47-2	0.021 ± 0.001	0.266 ± 0.007	0.244 ± 0.007 (1145.31 ± 30.50%)	2.40 × 10^{-4} (S)	517.18 ± 2.12	1152.99 ± 23.14	635.81 ± 17.32 (123.02 ± 3.71 %)	3.78 × 10^{-5} (HS)	Major Fe^{3+} interaction

Pinobanksin, a flavanonol (10)	548-82-3	0.025 ± 0.001	0.341 ± 0.003	0.315 ± 0.004 (1244.74 ± 15.95%)	5.47×10^{-5} (HS)	627.13 ± 20.07	1531.19 ± 21.45	904.06 ± 17.89 (144.29 ± 2.60 %)	6.58×10^{-5} (HS)	Major Fe^{3+} interaction
Taxifolin, a flavanonol (11)	480-18-2	0.042 ± 0.002	0.203 ± 0.008	0.161 ± 0.007 (386.40 ± 16.63%)	6.20×10^{-4} (S)	3349.67 ± 14.29	3759.60 ± 15.35	409.93 ± 10.81 (12.24 ± 0.33 %)	1.23×10^{-4} (S)	Some Fe^{3+} interaction
Catechin, a flavan-3-ol (12)	154-23-4	0.025 ± 0.001	0.318 ± 0.004	0.293 ± 0.004 (1173.33 ± 16.17%)	6.33×10^{-5} (HS)	4470.72 ± 12.93	5295.37 ± 25.44	824.65 ± 12.59 (18.45 ± 0.23 %)	7.77×10^{-5} (HS)	Major Fe^{3+} interaction
Epicatechin, a flavan-3-ol (13)	490-46-0	0.026 ± 0.002	0.445 ± 0.002	0.419 ± 0.001 (1633.77 ± 4.50%)	2.53×10^{-6} (HS)	4260.37 ± 93.59	5539.18 ± 98.74	1278.81 ± 6.21 (30.02 ± 0.55 %)	7.87×10^{-6} (HS)	Major Fe^{3+} interaction
Epigallocatechin, a flavan-3-ol (14)	970-74-1	0.032 ± 0.001	0.298 ± 0.003	0.266 ± 0.004 (832.29 ± 12.63%)	7.67×10^{-5} (HS)	5706.50 ± 15.22	6459.44 ± 19.94	752.94 ± 12.73 (13.19 ± 0.32 %)	1.65×10^{-4} (S)	Strong Fe^{3+} interaction
Formononetin, an isoflavone (15)	485-72-3	0.022 ± 0.002	0.290 ± 0.003	0.268 ± 0.002 (1218.18 ± 111.12%)	7.96×10^{-5} (HS)	940.26 ± 2.07	1186.12 ± 5.02	245.86 ± 2.95 (26.16 ± 0.33 %)	2.58×10^{-5} (HS)	Strong Fe^{3+} interaction
Genistein, an isoflavone (16)	446-72-0	0.023 ± 0.002	0.269 ± 0.002	0.246 ± 0.004 (1052.86 ± 15.05%)	6.81×10^{-5} (HS)	668.96 ± 7.85	1316.73 ± 7.98	647.77 ± 3.55 (96.83 ± 1.55 %)	4.56×10^{-5} (HS)	Strong Fe^{3+} interaction

Genistin, an isoflavone (17)	529-59-9	0.021 ± 0.002	0.036 ± 0.003	0.015 ± 0.002 (68.75 ± 9.76%)	6.65 × 10⁻³ (S)	576.94 ± 12.37	389.30 ± 14.12	-187.64 ± 7.55 (-32.52 ± 2.31 %)	1.02 × 10⁻³ (S)	No meaningful Fe^{3+} interference
Gallic Acid, a HBAD (18)	149-91-7	0.040 ± 0.001	0.026 ± 0.001	-0.014 ± 0.001 (-34.17 ± 1.44%)	5.90 × 10⁻⁴ (S)	7342.65 ± 97.23	7120.35 ± 98.54	-222.30 ± 6.74 (-3.03 ± 0.44 %)	9.12 × 10⁻⁴ (S)	No meaningful Fe^{3+} interference
Protocatechuic Acid, a HBAD (19)	99-50-3	0.034 ± 0.002	0.049 ± 0.002	0.015 ± 0.003 (44.55 ± 10.29%)	1.73 × 10⁻² (MS)	7264.97 ± 32.45	7122.47 ± 37.58	-142.50 ± 5.64 (-1.96 ± 0.11 %)	2.78 × 10⁻³ (S)	No meaningful Fe^{3+} interference
Methyl Syringate, a HBAD (20)	884-35-5	0.021 ± 0.001	0.022 ± 0.001	0.001 ± 0.002 (3.12 ± 7.16%)	5.29 × 10⁻¹ (NS)	3845.66 ± 2.81	3607.82 ± 2.17	-237.84 ± 2.49 (-6.18 ± 0.12 %)	7.55 × 10⁻³ (S)	No meaningful Fe^{3+} interference
Syringic Acid, a HBAD (21)	530-57-4	0.025 ± 0.001	0.061 ± 0.003	0.036 ± 0.004 (140.79 ± 15.95%)	4.25 × 10⁻³ (S)	4672.70 ± 7.21	4636.57 ± 5.98	-36.13 ± 2.45 (-0.77 ± 0.09 %)	1.99 × 10⁻² (MS)	No meaningful Fe^{3+} interference
2,3,4-THBA, a HBAD (22)	610-02-6	0.041 ± 0.001	0.046 ± 0.003	0.005 ± 0.003 (12.10 ± 7.26%)	1.02 × 10⁻¹ (NS)	7356.99 ± 110.46	7206.40 ± 116.80	-150.59 ± 9.02 (-2.05 ± 0.15 %)	1.19 × 10⁻³ (S)	No meaningful Fe^{3+} interference
Cinnamic Acid, a HCAD (23)	140-10-3	0.022 ± 0.001	0.023 ± 0.001	0.002 ± 0.002 (7.69 ± 10.66%)	3.38 × 10⁻¹ (NS)	341.49 ± 5.40	108.44 ± 7.92*	-233.05 ± 4.92 (-68.24 ± 2.54 %)	3.56 × 10⁻⁵ (HS)	No meaningful Fe^{3+} interference

Ferulic Acid, a HCAD (24)	537-98-4	0.025 ± 0.003	0.0303 ± 0.003	0.005 ± 0.005 (21.33 ± 18.04%)	1.77×10^{-1} (NS)	3954.97 ± 17.99	3741.68 ± 11.64	-213.29 ± 9.04 (-5.39 ± 0.23 %)	5.47×10^{-4} (S)	No meaningful Fe^{3+} interference
Rosmarinic Acid, a HCAD (25)	20283-92-5	0.041 ± 0.001	0.037 ± 0.004	-0.004 ± 0.004 (-10.40 ± 9.09%)	1.86×10^{-1} (NS)	7267.36 ± 12.88	7084.50 ± 16.89	-182.86 ± 7.38 (-2.52 ± 0.17 %)	2.01×10^{-3} (S)	No meaningful Fe^{3+} interference
m-Coumaric Acid, a HCAD (26)	14755-02-3	0.022 ± 0.001	0.021 ± 0.000	-0.001 ± 0.002 (-5.97 ± 6.84%)	2.70×10^{-1} (NS)	360.61 ± 3.29	119.20 ± 2.48*	-241.41 ± 2.39 (-66.96 ± 1.45 %)	4.99×10^{-5} (HS)	No meaningful Fe^{3+} interference
Lumichrome, a NP (27)	1086-80-2	0.023 ± 0.001	0.024 ± 0.001	0.001 ± 0.002 (5.88 ± 6.74%)	2.70×10^{-1} (NS)	420.37 ± 4.29	189.71 ± 1.98*	-230.66 ± 3.65 (-54.86 ± 1.75 %)	7.31×10^{-5} (HS)	No meaningful Fe^{3+} interference
Kojic Acid, a NP (28)	501-30-4	0.020 ± 0.001	0.047 ± 0.001	0.027 ± 0.001 (133.33 ± 2.89%)	1.6×10^{-4} (S)	294.88 ± 3.96	145.49 ± 2.87*	-149.39 ± 2.64 (-50.67 ± 1.89 %)	6.47×10^{-5} (HS)	Moderate Fe^{3+} binding

Legend: HBAD - hydroxybenzoic acid and derivatives; HCAD – hydroxycinnamic acid and derivatives; NP – non-phenolics; HS: highly significant; $p < 0.0001$; S: significant; $0.0001 \leq p < 0.01$; MS: marginally significant; $0.01 \leq p < 0.05$; NS: not significant; $p \geq 0.05$; Δ = difference

Table S3. Application of FRAP Assay in Analysing Western Australian Honeys and Comparison of Antioxidant Activity with and Without Honey Blanking (*n* = 3 per sample). FRAP Activity Expressed as mmol Fe/kg (Mean ± SD).

Honey	Code	FRAP w/ matrix-matched sample blank	FRAP w/ water blank	% Diff in FRAP	P value (t-test)
Blackbutt	BLA	4.21 ± 0.18	4.43 ± 0.15	5.23	0.179
Blackbutt	BLA	4.69 ± 0.07	4.90 ± 0.03	4.48	0.009*
Blackbutt	BLA	7.54 ± 0.42	7.73 ± 0.41	2.52	0.605
Blackbutt	BLA	6.55 ± 0.13	6.74 ± 0.15	2.90	0.173
Blackbutt	BLA	9.42 ± 0.18	9.69 ± 0.17	2.87	0.132
Blackbutt	BLA	6.35 ± 0.19	8.26 ± 0.23	30.08	<0.001*
Jarrah	JAR	7.87 ± 0.74	8.07 ± 0.72	2.54	0.754
Jarrah	JAR	7.83 ± 0.08	7.98 ± 0.04	1.92	0.044*
Jarrah	JAR	7.76 ± 0.45	7.94 ± 0.48	2.32	0.660
Jarrah	JAR	5.97 ± 0.19	6.09 ± 0.20	2.01	0.493
Jarrah	JAR	6.56 ± 0.22	6.68 ± 0.25	1.83	0.566
Jarrah	JAR	6.89 ± 0.28	7.10 ± 0.22	3.05	0.365
Jarrah	JAR	8.42 ± 0.41	10.86 ± 0.57	28.98	0.004*

Jarrah	JAR	5.38 ± 0.80	5.70 ± 0.15	5.94	0.533
Jarrah	JAR	7.49 ± 0.10	7.75 ± 0.09	3.42	0.029*
Jarrah	JAR	6.72 ± 0.24	7.21 ± 0.18	7.22	0.047*
Jarrah	JAR	4.30 ± 0.01	4.65 ± 0.06	8.07	<0.001*
Jarrah	JAR	4.76 ± 0.13	4.89 ± 0.14	2.63	0.304
Jarrah	JAR	4.35 ± 0.13	4.48 ± 0.15	2.92	0.320
Manuka	MAN	5.30 ± 0.04	5.52 ± 0.05	4.15	0.004*
Manuka	MAN	2.80 ± 0.12	3.02 ± 0.09	7.86	0.064
Manuka	MAN	3.97 ± 0.06	4.17 ± 0.05	5.04	0.011*
Manuka	MAN	5.45 ± 0.54	5.59 ± 0.51	2.57	0.760
Manuka	MAN	5.13 ± 0.17	5.27 ± 0.19	2.73	0.395
Manuka	MAN	5.43 ± 0.24	5.66 ± 0.23	4.24	0.297
Manuka	MAN	6.44 ± 0.16	6.58 ± 0.15	2.17	0.331
Marri	MAR	5.75 ± 0.11	6.01 ± 0.13	4.52	0.057
Marri	MAR	6.57 ± 0.47	6.71 ± 0.47	2.13	0.734
Marri	MAR	4.77 ± 0.08	5.00 ± 0.09	4.82	0.030*
Marri	MAR	5.10 ± 0.16	6.80 ± 0.24	33.33	<0.001*
Marri	MAR	3.96 ± 0.11	4.19 ± 0.06	5.70	0.034*

Multifloral	MUL	3.48 ± 0.05	3.65 ± 0.06	4.89	0.020*
Multifloral	MUL	3.00 ± 0.15	3.21 ± 0.08	7.00	0.099
Multifloral	MUL	6.80 ± 0.47	7.19 ± 0.46	5.74	0.362
Multifloral	MUL	2.81 ± 0.10	2.90 ± 0.10	3.20	0.332
Multifloral	MUL	5.19 ± 0.29	5.39 ± 0.32	3.85	0.467
Multifloral	MUL	2.99 ± 0.17	3.92 ± 0.17	31.10	0.003*
Multifloral	MUL	3.52 ± 0.04	4.58 ± 0.06	30.11	<0.001*
Multifloral	MUL	2.81 ± 0.09	3.73 ± 0.11	32.74	<0.001*
Multifloral	MUL	3.95 ± 0.07	5.09 ± 0.02	28.86	<0.001*
Multifloral	MUL	4.69 ± 0.13	4.76 ± 0.13	1.49	0.546
Multifloral	MUL	3.70 ± 0.33	3.84 ± 0.36	3.78	0.646
Multifloral	MUL	3.22 ± 0.07	3.44 ± 0.07	6.83	0.018*
Multifloral	MUL	9.52 ± 0.23	9.83 ± 0.21	3.23	0.160
Red Bell	RED	5.78 ± 0.27	5.84 ± 0.29	1.04	0.806
Red Bell	RED	5.54 ± 0.37	5.64 ± 0.37	1.81	0.757
Red Bell	RED	7.05 ± 0.23	7.31 ± 0.24	3.69	0.247

Figure S1. Chemical Structures of Phenolic Compounds and Related Antioxidants Tested in the FRAP Assay

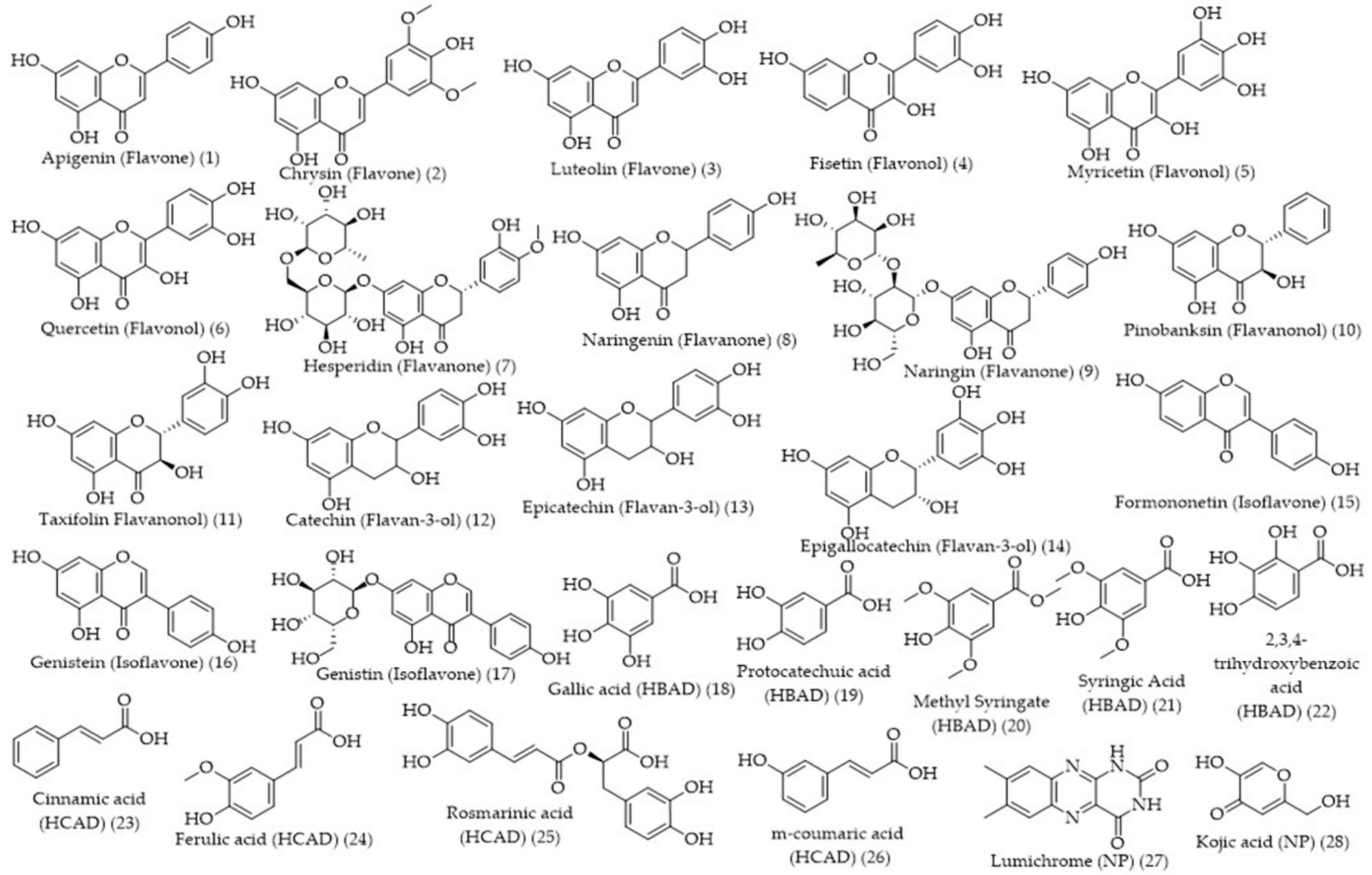

Chemical structures were generated using ChemDraw Version 25.0.2.14 (64-bit). Compound numbering corresponds to that used in Table S2 / main-text Table 4.

References

[2] Wu, J.; Xu, X.-T.; Xing, C.; Hao, X.-B.; Fang, X.-Y.; Xie, Z.-H.; Zhao, S.; Gao, J.-L.; Xu, L.; Wang, S.-J. Metabolic profiling and evaluation of antioxidant and anti-inflammatory properties of Apis cerana cerana Honey from Sansha City, Hainan Province, China. Food Chem. 2025, 475, 143256. https://doi.org/10.1016/j.foodchem.2025.143256.

[3] Fernandes, K.E.; Dong, A.Z.; Levina, A.; Cokcetin, N.N.; Brooks, P.; Carter, D.A. Long-term stability and the physical and chemical factors predictive for antimicrobial activity in Australian honey. PLoS ONE 2024, 19, e0303095. https://doi.org/10.1371/journal.pone.0303095.

[4] Kolayli, S.; Kazaz, G.; Özkök, A.; Keskin, M.; Kara, Y.; Demir Kanbur, E.; Ertürk, Ö. The phenolic composition, aroma compounds, physicochemical and antimicrobial properties of Nigella sativa L. (black cumin) honey. Eur. Food Res. Technol. 2023, 249, 653–664. https://doi.org/10.1007/s00217-022-04160-2.

[5] Ooi, T.C.; Yaacob, M.; Rajab, N.F.; Shahar, S.; Sharif, R. The stingless bee honey protects against hydrogen peroxide-induced oxidative damage and lipopolysaccharide-induced inflammation in vitro. Saudi J. Biol. Sci. 2021, 28, 2987–2994. https://doi.org/10.1016/j.sjbs.2021.02.039.

[6] Shamsi, T.; Azam, M.; Fatma, T.; Haque, Q.; Fatima, S.; Parveen, R.; Afreen, S. In-Vitro Antioxidant, Antibacterial And Anti-Inflammatory Characterization Of Indian Honey. Int. J. Pharm. Res. 2014, 8, 33–38.

[28] Beretta, G.; Granata, P.; Ferrero, M.; Orioli, M.; Maffei Facino, R. Standardization of antioxidant properties of honey by a combination of spectrophotometric/fluorimetric assays and chemometrics. Anal. Chim. Acta 2005, 533, 185–191. https://doi.org/10.1016/j.aca.2004.11.010.

[52] Laaroussi, H.; Bakour, M.; Ousaaid, D.; Ferreira-Santos, P.; Genisheva, Z.; El Ghouizi, A.; Aboulghazi, A.; Teixeira, J.A.; Lyoussi, B. Protective Effect of Honey and Propolis against Gentamicin-Induced Oxidative Stress and Hepatorenal Damages. Oxidative Med. Cell. Longev. 2021, 2021, 9719906. https://doi.org/10.1155/2021/9719906.

[53] Hunter, M.; McKune, A.; Toohey, K.; Naumovski, N. Interaction between properties of commercial Australian honey and saliva: In vitro exploratory study of a potential mechanism in the treatment of oral mucositis. J. Complement. Integr. Med. 2022, 19, 269–277. https://doi.org/10.1515/jcim-2021-0280.

[54] Cianciosi, D.; Forbes-Hernández, T.Y.; Giampieri, F.; Zhang, J.; Ansary, J.; Pacetti, M.; Quiles, J.L.; Simal-Gandara, J.; Battino, M. Effect of In vitro Gastrointestinal Digestion on the Bioaccessibility of Phenolic Compounds and Antioxidant Activity of Manuka Honey. eFood 2020, 1, 85–93. https://doi.org/10.2991/efood.k.191011.001.

[55] Perna, A.; Intaglietta, I.; Simonetti, A.; Gambacorta, E. Antioxidant activity of yogurt made from milk characterized by different casein haplotypes and fortified with chestnut and sulla honeys. J. Dairy Sci. 2014, 97, 6662–6670. https://doi.org/10.3168/jds.2013-7843.

[64] Mduda, C.A.; Rikohe, I.F.; Hussein, J.M.; Muruke, M.H. Characterization of Axestotrigona ferruginea and Apis mellifera honeys from different geographical origins. Food Humanit. 2025, 4, 100516. https://doi.org/10.1016/j.foohum.2025.100516.

[65] Morar, I.I.; Pop, R.M.; Peitzner, E.; Ranga, F.; Orăsan, M.S.; Cecan, A.D.; Chera, E.I.; Bonci, T.I.; Usatiuc, L.O.; Țicolea, M.; et al. Phytochemical Composition and Antioxidant Activity of Manuka Honey and Ohia Lehua Honey. Nutrients 2025, 17, 276. https://doi.org/10.3390/nu17020276.

[66] Dong, A.Z.; Cokcetin, N.; Carter, D.A.; Fernandes, K.E. Unique antimicrobial activity in honey from the Australian honeypot ant (Camponotus inflatus). PeerJ 2023, 11, e15645. https://doi.org/10.7717/peerj.15645.

[67] Mardhiah Mohd, S.; Mohd Alhafiizh, Z.; Wan Roslina Wan, Y.; Noorasmin Mokhtar, A. Effect of Thermal Treatment on Kelulut Honey Towards the Physicochemical, Antioxidant and Antimicrobial Properties. Borneo J. Resour. Sci. Technol. 2022, 12, 39–47. https://doi.org/10.33736/bjrst.4645.2022.

[68] Qi, N.; Zhao, W.; Xue, C.; Zhang, L.; Hu, H.; Jin, Y.; Xue, X.; Chen, R.; Zhang, J. Phenolic Acid and Flavonoid Content Analysis with Antioxidant Activity Assessment in Chinese C. pi. Shen Honey. Molecules 2025, 30, 370. https://doi.org/10.3390/molecules30020370.

[69] Puścion-Jakubik, A.; Bielecka, J.; Grabia, M.; Markiewicz-Żukowska, R.; Soroczyńska, J.; Teper, D.; Socha, K. Comparative Analysis of Antioxidant Properties of Honey from Poland, Italy, and Spain Based on the Declarations of Producers and Their Results of Melissopalinological Analysis. Nutrients 2022, 14, 2694. https://doi.org/10.3390/nu14132694.

[70] Qahtani, H.; Yagi, S.; Yilmaz, M.; Çakır, O.; Tarhan, A.; Ali Mustafa, A.; Zengin, G. Chemical Profile, Antioxidant and Enzyme Inhibition Activities of Natural Saudi Sidr and Talh Honeys. Chem. Biodivers. 2022, 19, e202200227. https://doi.org/10.1002/cbdv.202200227.

[71] Moein, M.; Moein, S.; Farmani, F.; Rozbehan, S.; Sabahi, Z. Examination the antioxidant potentials and antidiabetic properties of phenolic extracts of some Iranian honeys. J. Nephropharmacol 2022, 11, e6. https://doi.org/10.34172/npj.2022.06.

[72] Mat Ramlan, N.A.F.; Md Zin, A.S.; Safari, N.F.; Chan, K.W.; Zawawi, N. Application of Heating on the Antioxidant and Antibacterial Properties of Malaysian and Australian Stingless Bee Honey. Antibiotics 2021, 10, 1365. https://doi.org/10.3390/antibiotics10111365.

[73] Kek, S.P.; Chin, N.L.; Yusof, Y.A.; Tan, S.W.; Chua, L.S. Classification of entomological origin of honey based on its physico-chemical and antioxidant properties. Int. J. Food Prop. 2017, 20, S2723–S2738. https://doi.org/10.1080/10942912.2017.1359185.

[74] Shamsudin, S.; Selamat, J.; Abdul Shomad, M.; Ab Aziz, M.F.; Haque Akanda, M.J. Antioxidant Properties and Characterization of Heterotrigona itama Honey from Various Botanical Origins according to Their Polyphenol Compounds. J. Food Qual. 2022, 2022, 2893401. https://doi.org/10.1155/2022/2893401.

[75] Zhu, M.; Zhao, H.; Wang, Q.; Wu, F.; Cao, W. A Novel Chinese Honey from Amorpha fruticosa L.: Nutritional Composition and Antioxidant Capacity In Vitro. Molecules 2020, 25, 5211. https://doi.org/10.3390/molecules25215211.

[76] Zhao, H.; Cheng, N.; He, L.; Peng, G.; Xue, X.; Wu, L.; Cao, W. Antioxidant and hepatoprotective effects of A. cerana honey against acute alcohol-induced liver damage in mice. Food Res. Int. 2017, 101, 35–44. https://doi.org/10.1016/j.foodres.2017.08.014.

[77] Cheng, N.; Du, B.; Wang, Y.; Gao, H.; Cao, W.; Zheng, J.; Feng, F. Antioxidant properties of jujube honey and its protective effects against chronic alcohol-induced liver damage in mice. Food Funct. 2014, 5, 900–908. https://doi.org/10.1039/C3FO60623F.

[78] Cheng, N.; Wu, L.; Zheng, J.; Cao, W. Buckwheat Honey Attenuates Carbon Tetrachloride-Induced Liver and DNA Damage in Mice. Evid.-Based Complement. Altern. Med. 2015, 2015, 987385. https://doi.org/10.1155/2015/987385.

[79] Lianda, R.; Sant'Ana, L.; Echevarria, A.; Nora Castro, R. Antioxidant Activity and Phenolic Composition of Brazilian Honeys and their Extracts. J. Braz. Chem. Soc. 2012, 23, 618–627. https://doi.org/10.1590/S0103-50532012000400006.

[80] Turkut, G.M.; Degirmenci, A.; Yildiz, O.; Can, Z.; Cavrar, S.; Yaylaci Karahalil, F.; Kolayli, S. Investigating 5-hydroxymethylfurfural formation kinetic and antioxidant activity in heat treated honey from different floral sources. J. Food Meas. Charact. 2018, 12, 2358–2365. https://doi.org/10.1007/s11694-018-9852-y.

[81] Kolayli, S.; Can, Z.; Çakir, H.E.; Okan, O.T.; Yildiz, O. An investigation on Trakya region Oak (Quercus spp.) honeys of Turkey: Their physico-chemical, antioxidant and phenolic compounds properties. Turk. J. Biochem. 2018, 43, 362–374. https://doi.org/10.1515/tjb-2017-0174.

[82] Starowicz, M.; Ostaszyk, A.; Zieliński, H. The Relationship between the Browning Index, Total Phenolics, Color, and Antioxidant Activity of Polish-Originated Honey Samples. Foods 2021, 10, 967. https://doi.org/10.3390/foods10050967.

[83] Zarei, M.; Fazlara, A.; Alijani, N. Evaluation of the changes in physicochemical and antioxidant properties of honey during storage. Funct. Foods Health Dis. 2019, 9, 593–605. https://doi.org/10.31989/ffhd.v9i9.616.

[84] Alkan, S.; Akgün, M.; Ertürk, Ö.; Çol Ayvaz, M.; Başkan, C. Properties of Honey and Pollen Samples Obtained from Different Rhododendron Species Collected from Black Sea Region of Turkey. J. Apic. Sci. 2020, 64, 321–334. https://doi.org/10.2478/jas-2020-0028.

[85] Otmani, A.; Ouchemoukh, N.; Mouhoubi-Tafinine, Z.; Tighlit, K.; Redouan, I.; Terrab, A.; Ouchemoukh, S. Contribution of organic bee pollen to the determination of the botanical origin of honey and its impact on its biological properties. Curr. Bioact. Compd. 2021, 18, e301221199687. https://doi.org/10.2174/1573407218666211230150151.

[86] Zivkovic, J.; Sunaric, S.; Stanković, N.; Mihajilov-Krstev, T.; Ana, S. Total phenolic and flavonoid contents, antioxidant and antibacterial activities of selected honeys against human pathogenic bacteria. Acta Pol. Pharm. 2019, 67, 671–681. https://doi.org/10.32383/appdr/105461.

[87] Kulkarni, S.S.; Mishra, S.; Patil, S.B.; Nambiar, J.; Math, A. Unifloral ajwain honey ameliorates differential inhibition of matrix metalloproteinases 2 and 9 protein, cytotoxicity, and antioxidant potential. J. Ayurveda Integr. Med. 2021, 12, 633–639. https://doi.org/10.1016/j.jaim.2021.05.012.

[88] Wan Yaacob, W.; Long, I.; Zakaria, R.; Othman, Z. Investigation of antioxidant potentials of methanolic and ethyl acetate fractions of Tualang honey. *Int. Med. J.* **2020**, *27*, 748–751. https://doi.org/10.5281/zenodo.5152400

[89] Gorjanović, S.Ž.; Alvarez-Suarez, J.M.; Novaković, M.M.; Pastor, F.T.; Pezo, L.; Battino, M.; Sužnjević, D.Ž. Comparative analysis of antioxidant activity of honey of different floral sources using recently developed polarographic and various spectrophotometric assays. J. Food Compos. Anal. 2013, 30, 13–18. https://doi.org/10.1016/j.jfca.2012.12.004.

[90] Mohammadi, Y.; Tahergorabi, Z.; Sharifzadeh, G.R.; Rajabi Moghadam, M.; Zarban, A. Protective Effects of Some Graded Iranian Honey Samples Against Cold Water Immersion-Induced Gastric Ulcers in Rats. Food Sci. Nutr. 2024, 12, 10211–10222. https://doi.org/10.1002/fsn3.4567.

[91] Alarjani, W.M.A.; Mohammed, M.E.A. Antioxidant activities of Saudi honey samples related to their content of short peptides. Sci. Rep. 2024, 14, 24318. https://doi.org/10.1038/s41598-024-74824-4.

[92] Kolayli, S.; Can, Z.; Kara, Y.; Ozkok, A.; Ozmert Ergin, S.; Kemal, M.; Demir Kanbur, E. Physicochemical Characteristics, Phenolic Components, and Antioxidant Capacities of Lavender Honey (Lavandula spp.) from Isparta Region of Türkiye. Chem. Biodivers. 2024, 21, e202400718. https://doi.org/10.1002/cbdv.202400718.

[93] Hajian-Tilaki, A.; Kenari, R.E.; Razavi, R.; Farahmandfar, R. Phenolic profile, antioxidant properties, and pollen spectra of Iranian-originated honeys. Eur. Food Res. Technol. 2024, 250, 2317–2329. https://doi.org/10.1007/s00217-024-04539-3.

[94] Mduda, C.A.; Muruke, M.H.; Joseph, C.O.; Hussein, J.M. Antioxidant and antibacterial properties of stingless bee (Meliponula spp.) honey from the northern highlands of Tanzania, in comparison with Apis mellifera honey. Food Humanit. 2024, 2, 100310. https://doi.org/10.1016/j.foohum.2024.100310.

[95] Yaylaci Karahalil, F.; Bakirhan, F.; Kara, Y. In Vitro Determination of Nitric Oxide Synthase Inhibition, Antioxidant Capacity and Phenolic Content of Various Natural Products (Bee and Herbal Products). Chem. Biodivers. 2024, 21, e202301330. https://doi.org/10.1002/cbdv.202301330.

[96] Harbane, S.; Escuredo, O.; Saker, Y.; Ghorab, A.; Nakib, R.; Rodríguez-Flores, M.S.; Ouelhadj, A.; Seijo, M.C. The Contribution of Botanical Origin to the Physicochemical and Antioxidant Properties of Algerian Honeys. Foods 2024, 13, 573. https://doi.org/10.3390/foods13040573.

[97] Mduda, C.A.; Hussein, J.M.; Muruke, M.H. The effects of bee species and vegetation on the antioxidant properties of honeys produced by Afrotropical stingless bees (Hymenoptera, Apidae, Meliponini). J. Agric. Food Res. 2023, 14, 100736. https://doi.org/10.1016/j.jafr.2023.100736.

[98] Ismail, M.; Abdallah, E.; Musa, K.H.; Abdellatif, A.A.H. First report of some chemical and biological properties of two wild honey types collected from a remote area in Southern Darfur, Sudan. Food Res. 2023, 7, 360. https://doi.org/10.26656/fr.2017.7(3).360.

[99] Kemal, M.; Esertaş, Ü.Z.Ü.; Kanbur, E.D.; Kara, Y.; Özçelik, A.E.; Can, Z.; Kolaylı, S. Characterization of the black cumin (Nigella sativa L.) honey from Türkiye. Food Biosci. 2023, 53, 102760. https://doi.org/10.1016/j.fbio.2023.102760.

[100] Uçar, M.; Kemal, M.; Kanbur, E.D.; Kara, Y.; Özcelik, A.E.; Kolaylı, S. The botanical, physicochemical, and biochemical characteristics of Northern Cyprus honeys. Eur. Food Res. Technol. 2023, 249, 1531–1541. https://doi.org/10.1007/s00217-023-04233-w.

[101] Saral, Ö. An Investigation into Chestnut Honeys from Artvin Province in Turkiye: Their Physicochemical Properties, Phenolic Profiles and Antioxidant Activities. Chem. Biodivers. 2023, 20, e202201162. https://doi.org/10.1002/cbdv.202201162.

[102] Fernández-Estellé, M.; Hernández-González, V.; Saurina, J.; Núñez, O.; Sentellas, S. Characterization and Classification of Spanish Honeydew and Blossom Honeys Based on Their Antioxidant Capacity. Antioxidants 2023, 12, 495. https://doi.org/10.3390/antiox12020495.

[103] Sahin, H.; Ozkok, A.; Tanugur Samanci, A.E.; Onder, E.Y.; Kolayli, S. Identification of the Main Phenolic Markers in Turkish Pine Honeys and Their Biological Functions. Chem. Biodivers. 2022, 19, e202200835. https://doi.org/10.1002/cbdv.202200835.

[104] Jurič, A.; Huđek Turković, A.; Brčić Karačonji, I.; Prđun, S.; Bubalo, D.; Durgo, K. Cytotoxic activity of strawberry tree (Arbutus unedo L.) honey, its extract, and homogentisic acid on CAL 27, HepG2, and Caco-2 cell lines. Arch. Ind. Hyg. Toxicol. 2022, 73, 158–168. https://doi.org/10.2478/aiht-2022-73-3653.

[105] Wu, J.; Han, B.; Zhao, S.; Zhong, Y.; Han, W.; Gao, J.; Wang, S. Bioactive characterization of multifloral honeys from Apis cerana cerana, Apis dorsata, and Lepidotrigona flavibasis. Food Res. Int. 2022, 161, 111808. https://doi.org/10.1016/j.foodres.2022.111808.

[106] Balkanska, R.; Shumkova, R. Preliminary study of antioxidant activity of polyfloral and sunflower honey from Bulgaria. Bulg. J. Agric. Sci. 2022, 28, 764–767. https://agriacad.eu/ojs/index.php/bjas/article/view/1672 (accessed on May 4, 2026)

[107] Bouacha, M.; Besnaci, S.; Boudiar, I.; Al-Kafaween, M.A. Impact of Storage on Honey Antibacterial and Antioxidant Activities and their Correlation with Polyphenolic Content. Trop. J. Nat. Product. Res. (TJNPR) 2022, 6, 34–39. https://doi.org/10.26538/tjnpr/v6i1.7.

[108] Lazarus, M.; Tariba Lovaković, B.; Sekovanić, A.; Orct, T.; Jurič, A.; Prđun, S.; Denžić Lugomer, M.; Bubalo, D. Combined approach to studying authenticity markers following spatial, temporal and production practice trends in honey from Croatia. Eur. Food Res. Technol. 2021, 247, 1511–1523. https://doi.org/10.1007/s00217-021-03728-8.

[109] Ng, W.-J.; Sit, N.-W.; Ooi, P.A.-C.; Ee, K.-Y.; Lim, T.-M. Botanical Origin Differentiation of Malaysian Stingless Bee Honey Produced by Heterotrigona itama and Geniotrigona thoracica Using Chemometrics. Molecules 2021, 26, 7628. https://doi.org/10.3390/molecules26247628.

[110] Olate-Olave, V.R.; Guzmán, L.; López-Cortés, X.A.; Cornejo, R.; Nachtigall, F.M.; Doorn, M.; Santos, L.S.; Bejarano, A. Comparison of Chilean honeys through MALDI-TOF-MS profiling and evaluation of their antioxidant and antibacterial potential. Ann. Agric. Sci. 2021, 66, 152–161. https://doi.org/10.1016/j.aoas.2021.11.001.

[111] Ganaie, T.A.; Masoodi, F.A.; Rather, S.A.; Wani, S.M. Physicochemical, antioxidant and FTIR-ATR spectroscopy evaluation of Kashmiri honeys as food quality traceability and Himalayan brand. J. Food Sci. Technol. 2021, 58, 4139–4148. https://doi.org/10.1007/s13197-020-04878-5.

[112] Ara, C.; Asmatullah; Yaseen, F.; Ali, S.; Shakir, H.A.; Khan, M.; Andleeb, S.; Ramzan, N. Evaluation of sex steroid hormones and reproductive irregularities in diethyl phthalate-exposed premature mice: Modulatory effect of raw honey against potential anomalies. Environ. Sci. Pollut. Res. 2021, 28, 55265–55276. https://doi.org/10.1007/s11356-021-14774-w.

[113] Kędzierska-Matysek, M.; Teter, A.; Stryjecka, M.; Skałecki, P.; Domaradzki, P.; Rudaś, M.; Florek, M. Relationships Linking the Colour and Elemental Concentrations of Blossom Honeys with Their Antioxidant Activity: A Chemometric Approach. Agriculture 2021, 11, 702. https://doi.org/10.3390/agriculture11080702.

[114] Cacique, A.P.; Barbosa, É.S.; de Pinho, G.P.; Silvério, F.O. Miniaturized Methodologies for Determining the Total Phenol and Flavonoid Concentrations and the Antioxidant Activity. Food Anal. Methods 2021, 14, 1110–1120. https://doi.org/10.1007/s12161-020-01934-w.

[115] Majkut, M.; Kwiecińska-Piróg, J.; Wszelaczyńska, E.; Pobereżny, J.; Gospodarek-Komkowska, E.; Wojtacki, K.; Barczak, T. Antimicrobial activity of heat-treated Polish honeys. Food Chem. 2021, 343, 128561. https://doi.org/10.1016/j.foodchem.2020.128561.

[116] Chen, Y.H.; Chuah, W.C.; Chye, F.Y. Effect of drying on physicochemical and functional properties of stingless bee honey. J. Food Process. Preserv. 2021, 45, e15328. https://doi.org/10.1111/jfpp.15328.

[117] Mahmood, A.L.; Lani, M.; Hassan, Z.; Abd Razak, S.B.; Ahmad, F.T. Antioxidant and antimicrobial properties of Indo-Malayan stingless bee (Heterotrigona itama) honey from different seasons and distribution of flowers. Food Res. 2021, 5, 498–507. https://doi.org/10.26656/fr.2017.5(2).546.

[118] Pentoś, K.; Łuczycka, D.; Oszmiański, J.; Lachowicz, S.; Pasternak, G. Polish honey as a source of antioxidants—A comparison with Manuka honey. J. Apic. Res. 2020, 59, 939–945. https://doi.org/10.1080/00218839.2020.1723837.

[119] Degirmenci, A.; Can, Z.; Boyraci, G.M.; Yildiz, O.; Asadov, E.; Kolayli, S. Honeys from some different regions of Azerbaijan: Bioactive characteristics based on phenolic profile and antioxidant activity. J. Apic. Res. 2020, 59, 390–397. https://doi.org/10.1080/00218839.2020.1726033.

[120] Ibrahimi, H.; Hajdari, A. Phenolic and flavonoid content, and antioxidant activity of honey from Kosovo. J. Apic. Res. 2020, 59, 452–457. https://doi.org/10.1080/00218839.2020.1714194.

[121] Missio da Silva, P.; Gonzaga, L.V.; Biluca, F.C.; Schulz, M.; Vitali, L.; Micke, G.A.; Oliveira Costa, A.C.; Fett, R. Stability of Brazilian Apis mellifera L. honey during prolonged storage: Physicochemical parameters and bioactive compounds. LWT 2020, 129, 109521. https://doi.org/10.1016/j.lwt.2020.109521.

[122] Nayaka, N.M.D.M.W.; Fidrianny, I.; Sukrasno; Hartati, R.; Singgih, M. Antioxidant and antibacterial activities of multiflora honey extracts from the Indonesian Apis cerana bee. J. Taibah Univ. Med. Sci. 2020, 15, 211–217. https://doi.org/10.1016/j.jtumed.2020.04.005.

[123] Erturk, Ö.; Kalın, S.; Ayvaz, M.Ç. Physicochemical properties, bioactive components, antioxidant and antimicrobial potentials of some selected honeys from different provinces of Turkey. Br. Food J. 2019, 121, 1298–1313. https://doi.org/10.1108/BFJ-04-2018-0261.

[124] Zarei, M.; Fazlara, A.; Tulabifard, N. Effect of thermal treatment on physicochemical and antioxidant properties of honey. Heliyon 2019, 5, e01894. https://doi.org/10.1016/j.heliyon.2019.e01894.

[125] Tuksitha, L.; Chen, Y.-L.S.; Chen, Y.-L.; Wong, K.-Y.; Peng, C.-C. Antioxidant and antibacterial capacity of stingless bee honey from Borneo (Sarawak). J. Asia-Pac. Entomol. 2018, 21, 563–570. https://doi.org/10.1016/j.aspen.2018.03.007.

[126] Perna, A.; Simonetti, A.; Gambacorta, E. Phenolic content and antioxidant activity of donkey milk kefir fortified with sulla honey and rosemary essential oil during refrigerated storage. Int. J. Dairy Technol. 2019, 72, 74–81. https://doi.org/10.1111/1471-0307.12561.

[127] Malkoç, M.; Çakır, H.; Kara, Y.; Can, Z.; Kolaylı, S. Phenolic Composition and Antioxidant Properties of Anzer Honey from Black Sea Region of Turkey. Uludag Bee J. 2019, 19, 143–151. https://doi.org/10.31467/uluaricilik.602906.

[128] Saral, O.; KiliÇArslan, M.; ŞAhİN, H.; Yildiz, O.; Dincer, B. Evaluation of antioxidant activity of bee products of different bee races in Turkey. Turk. J. Vet. Anim. Sci. 2019, 43, 441–447. https://doi.org/10.3906/vet-1901-3.

[129] Chaiyasut, C.; Kesika, P.; Peerajan, S.; Sivamaruthi, B.S. The Total Polyphenolic Content and Antioxidant Properties of Various Honey and Sugars. Asian J. Pharm. Clin. Res. 2018, 11, 467–471. https://doi.org/10.22159/ajpcr.2018.v11i5.24696.

[130] Nascimento, K.S.D.; Gasparotto Sattler, J.A.; Lauer Macedo, L.F.; Serna González, C.V.; Pereira de Melo, I.L.; da Silva Araújo, E.; Granato, D.; Sattler, A.; de Almeida-Muradian, L.B. Phenolic compounds, antioxidant capacity and physicochemical properties of Brazilian Apis mellifera honeys. LWT 2018, 91, 85–94. https://doi.org/10.1016/j.lwt.2018.01.016.

[131] Moloudian, H.; Abbasian, S.; Nassiri-Koopaei, N.; Tahmasbi, M.R.; Alsadat Afzal, G.; Ahosseini, M.S.; Yunesian, M.; Khoshayand, M.R. Characterization and Classification of Iranian Honey Based on Physicochemical Properties and Antioxidant Activities, with Chemometrics Approach. Iran. J. Pharm. Res. 2018, 17, 708–725.

[132] Grabek-Lejko, D.; Słowik, J.; Kasprzyk, I. Activity of selected honey types against Staphylococcus aureus methicillin susceptible (MSSA) and methicillin resistant (MRSA) bacteria and its correlation with hydrogen peroxide, phenolic content and antioxidant capacity. Rev. Farm. 2018, 66, 37–43.

[133] de Oliveira Neto, J.R.; Rezende, S.G.; Lobón, G.S.; Garcia, T.A.; Macedo, I.Y.L.; Garcia, L.F.; Alves, V.F.; Torres, I.M.S.; Santiago, M.F.; Schmidt, F.; et al. Electroanalysis and laccase-based

biosensor on the determination of phenolic content and antioxidant power of honey samples. Food Chem. 2017, 237, 1118–1123. https://doi.org/10.1016/j.foodchem.2017.06.010.

[134] Nweze, J.A.; Okafor, J.I.; Nweze, E.I.; Nweze, J.E. Evaluation of physicochemical and antioxidant properties of two stingless bee honeys: A comparison with Apis mellifera honey from Nsukka, Nigeria. BMC Res. Notes 2017, 10, 566. https://doi.org/10.1186/s13104-017-2884-2.

[135] Petretto, G.L.; Tuberoso, C.I.G.; Fenu, M.A.; Rourke, J.P.; Belhaj, O.; Pintore, G. Antioxidant activity, color chromaticity coordinates, and chemical characterization of monofloral honeys from Morocco. Int. J. Food Prop. 2017, 20, 2016–2027. https://doi.org/10.1080/10942912.2016.1230745.

[136] Kuś, P.M.; Jerković, I.; Marijanović, Z.; Tuberoso, C.I.G. Screening of Polish Fir Honeydew Honey Using GC/MS, HPLC-DAD, and Physical-Chemical Parameters: Benzene Derivatives and Terpenes as Chemical Markers. Chem. Biodivers. 2017, 14, e1700179. https://doi.org/10.1002/cbdv.201700179.

[137] Soares, S.; Pinto, D.; Rodrigues, F.; Alves, R.C.; Oliveira, M.B.P.P. Portuguese Honeys from Different Geographical and Botanical Origins: A 4-Year Stability Study Regarding Quality Parameters and Antioxidant Activity. Molecules 2017, 22, 1338. https://doi.org/10.3390/molecules22081338.

[138] Sowa, P.; Grabek-Lejko, D.; Wesołowska, M.; Swacha, S.; Dżugan, M. Hydrogen peroxide-dependent antibacterial action of Melilotus albus honey. Lett. Appl. Microbiol. 2017, 65, 82–89. https://doi.org/10.1111/lam.12749.

[139] Afrin, S.; Forbes-Hernandez, T.Y.; Gasparrini, M.; Bompadre, S.; Quiles, J.L.; Sanna, G.; Spano, N.; Giampieri, F.; Battino, M. Strawberry-Tree Honey Induces Growth Inhibition of Human Colon Cancer Cells and Increases ROS Generation: A Comparison with Manuka Honey. Int. J. Mol. Sci. 2017, 18, 613. https://doi.org/10.3390/ijms18030613.

[140] Jerković, I.; Marijanović, Z.; Zekić, M.; Tuberoso, C.I.G. First Report on Rare Unifloral Honey of Endemic Moltkia petraea (Tratt.) Griseb. from Croatia: Detailed Chemical Screening and Antioxidant Capacity. Chem. Biodivers. 2017, 14, e1600268. https://doi.org/10.1002/cbdv.201600268.

[141] Hagr, T.; Mirghani, M.; Elnour, A.; Bkharsa, B. Antioxidant capacity and sugar content of honey from Blue Nile State, Sudan. Int. Food Res. J. 2017, 24, 452–456.

[142] Ahmed, M.; Imtiaz Shafiq, M.; Khaleeq, A.; Huma, R.; Abdul Qadir, M.; Khalid, A.; Ali, A.; Samad, A. Physiochemical, Biochemical, Minerals Content Analysis, and Antioxidant Potential of National and International Honeys in Pakistan. J. Chem. 2016, 2016, 8072305. https://doi.org/10.1155/2016/8072305.

[143] Kolayli, S.; Can, Z.; Yildiz, O.; Sahin, H.; Karaoglu, S.A. A comparative study of the antihyaluronidase, antiurease, antioxidant, antimicrobial and physicochemical properties of different unifloral degrees of chestnut (Castanea sativa Mill.) honeys. J. Enzym. Inhib. Med. Chem. 2016, 31, 96–104. https://doi.org/10.1080/14756366.2016.1209494.

[144] Sahin, H. Honey as an apitherapic product: Its inhibitory effect on urease and xanthine oxidase. J. Enzym. Inhib. Med. Chem. 2016, 31, 490–494. https://doi.org/10.3109/14756366.2015.1039532.

[145] Bundit, T.; Anothai, T.; Pattaramart, P.; Roongpet, T.; Chuleeporn, S. Comparison of antioxidant contents of Thai Honeys to Manuka Honey. Malays. J. Nutr. 2016, 22, 413–420.

[146] Sahin, H.; Turumtay, E.A.; Yildiz, O.; Kolayli, S. Grayanotoxin-III Detection and Antioxidant Activity of Mad Honey. Int. J. Food Prop. 2015, 18, 2665–2674. https://doi.org/10.1080/10942912.2014.999866.

[147] Khalil, M.I.; Islam, M.A.; Alam, N.; Gan, S.H.; Sulaiman, S.A. Irradiation and Evaporation Enhance Physicochemical Characteristics, AEAC, FRAP, Protein and Proline Contents of Tualang Honey. J. Food Biochem. 2015, 39, 742–753. https://doi.org/10.1111/jfbc.12182.

[148] Jerković, I.; Tuberoso, C.I.G.; Marijanović, Z.; Kranjac, M.; Malenica-Staver, M. Antioxidant Capacity and Chemical Profiles of Satureja montana L. Honey: Hotrienol and Syringyl Derivatives as Biomarkers. Chem. Biodivers. 2015, 12, 1047–1056. https://doi.org/10.1002/cbdv.201400183.

[149] Wang, Y.; Li, D.; Cheng, N.; Gao, H.; Xue, X.; Cao, W.; Sun, L. Antioxidant and hepatoprotective activity of vitex honey against paracetamol induced liver damage in mice. Food Funct. 2015, 6, 2339–2349. https://doi.org/10.1039/C5FO00345H.

[150] Das, A.; Datta, S.; Mukherjee, S.; Bose, S.; Ghosh, S.; Dhar, P. Evaluation of antioxidative, antibacterial and probiotic growth stimulatory activities of Sesamum indicum honey containing phenolic compounds and lignans. LWT-Food Sci. Technol. 2015, 61, 244–250. https://doi.org/10.1016/j.lwt.2014.11.044.

[151] Ng, W.-J.; Lim, M.-S. Anti-staphylococcal activity of Melaleuca honey. Southeast Asian J. Trop. Med. Public Health 2015, 46, 472–479.

[152] Yap, P.; Abu Bakar, M.F. Antioxidative and acetylcholinesterase inhibitor potential of selected honey of Sabah, Malaysian Borneo. Int. Food Res. J. 2015, 22, 1953–1960.

[153] Yildiz, O.; Karahalil, F.; Can, Z.; Sahin, H.; Kolayli, S. Total monoamine oxidase (MAO) inhibition by chestnut honey, pollen and propolis. J. Enzym. Inhib. Med. Chem. 2014, 29, 690–694. https://doi.org/10.3109/14756366.2013.843171.

[154] Dor, G.O.L.M.; Mahomoodally, M.F. Chemical profile and in vitro bioactivity of tropical honey from Mauritius. Asian Pac. J. Trop. Dis. 2014, 4, S1002–S1013. https://doi.org/10.1016/S2222-1808(14)60773-8.

[155] Gambacorta, E.; Simonetti, A.; Garrisi, N.; Intaglietta, I.; Perna, A. Antioxidant properties and phenolic content of sulla (Hedysarum spp.) honeys from Southern Italy. Int. J. Food Sci. Technol. 2014, 49, 2260–2268. https://doi.org/10.1111/ijfs.12541.

[156] Moniruzzaman, M.; Yung An, C.; Rao, P.V.; Hawlader, M.N.I.; Azlan, S.A.B.M.; Sulaiman, S.A.; Gan, S.H. Identification of Phenolic Acids and Flavonoids in Monofloral Honey from Bangladesh by High Performance Liquid Chromatography: Determination of Antioxidant Capacity. BioMed Res. Int. 2014, 2014, 737490. https://doi.org/10.1155/2014/737490.

[157] Ertürk, O.; Sahin, H.; Kolayli, S.; Çol Ayvaz, M. Antioxidant and antimicrobial activity of East Black Sea Region honeys. Turk. J. Biochem. 2014, 39, 99–106. https://doi.org/10.5505/tjb.2014.77487.

[158] Kuś, P.M.; Congiu, F.; Teper, D.; Sroka, Z.; Jerković, I.; Tuberoso, C.I.G. Antioxidant activity, color characteristics, total phenol content and general HPLC fingerprints of six Polish unifloral honey types. LWT-Food Sci. Technol. 2014, 55, 124–130. https://doi.org/10.1016/j.lwt.2013.09.016.

[159] Kuś, P.M.; Jerković, I.; Tuberoso, C.I.G.; Marijanović, Z.; Congiu, F. Cornflower (Centaurea cyanus L.) honey quality parameters: Chromatographic fingerprints, chemical biomarkers, antioxidant capacity and others. Food Chem. 2014, 142, 12–18. https://doi.org/10.1016/j.foodchem.2013.07.050.

[160] Chua, L.S.; Rahaman, N.L.A.; Adnan, N.A.; Eddie Tan, T.T. Antioxidant Activity of Three Honey Samples in relation with Their Biochemical Components. J. Anal. Methods Chem. 2013, 2013, 313798. https://doi.org/10.1155/2013/313798.

[161] Maksimović, Z.; Nedic, N. In vitro antioxidant activity of honeydew and multifloral types of honey from Serbia. Acta Period. Technol. 2013, 44, 269. https://doi.org/10.2298/APT1344269M.

[162] Moniruzzaman, M.; Sulaiman, S.A.; Azlan, S.A.M.; Gan, S.H. Two-Year Variations of Phenolics, Flavonoids and Antioxidant Contents in Acacia Honey. Molecules 2013, 18, 14694–14710. https://doi.org/10.3390/molecules181214694.

[163] Grabek-Lejko, D.; Tomczyk-Ulanowska, K. Phenolic content, antioxidant and antibacterial activity of selected natural sweeteners available on the Polish market. J. Environ. Sci. Health Part B 2013, 48, 1089–1096. https://doi.org/10.1080/03601234.2013.824236.

[164] Jubri, Z.; Rahim, N.B.A.; Aan, G.J. Manuka honey protects middle-aged rats from oxidative damage. Clinics 2013, 68, 1446–1454. https://doi.org/10.6061/clinics/2013(11)11.

[165] Tuberoso, C.I.G.; Boban, M.; Bifulco, E.; Budimir, D.; Pirisi, F.M. Antioxidant capacity and vasodilatory properties of Mediterranean food: The case of Cannonau wine, myrtle berries liqueur and strawberry-tree honey. Food Chem. 2013, 140, 686–691. https://doi.org/10.1016/j.foodchem.2012.09.071.

[166] Srimeena, N.; Gunasekaran, S.; Murugesan, R.; Muthuraman, M. Evaluation of Antioxidant Activity and Characterization of Indian Rock Bee Mead. J. Pure Appl. Microbiol. 2013, 7, 3185–3190.

[167] Moniruzzaman, M.; Sulaiman, S.A.; Khalil, M.I.; Gan, S.H. Evaluation of physicochemical and antioxidant properties of sourwood and other Malaysian honeys: A comparison with manuka honey. Chem. Cent. J. 2013, 7, 138. https://doi.org/10.1186/1752-153X-7-138.

[168] Schneider, M.; Coyle, S.; Warnock, M.; Gow, I.; Fyfe, L. Anti-Microbial Activity and Composition of Manuka and Portobello Honey. Phyther. Res. 2013, 27, 1162–1168. https://doi.org/10.1002/ptr.4844.

[169] Šarić, G.M.; Vukićević, K.; Lež, D.; Hruškar, E.; Vahčić, M. Changes of antioxidant activity in honey after heat treatment. Czech. J. Food Sci. 2013, 31, 601–606.

[170] Islam, A.; Khalil, I.; Islam, N.; Moniruzzaman, M.; Mottalib, A.; Sulaiman, S.A.; Gan, S.H. Physicochemical and antioxidant properties of Bangladeshi honeys stored for more than one year. BMC Complement. Altern. Med. 2012, 12, 177. https://doi.org/10.1186/1472-6882-12-177.

[171] Šarić, G.; Marković, K.; Major, N.; Krpan, M.; Uršulin-Trstenjak, N.; Hruskar, M.; Vahcić, N. Changes of Antioxidant Activity and Phenolic Content in Acacia and Multifloral Honey During Storage. Food Technol. Biotechnol. 2012, 50, 434–441.

[172] Perna, A.; Simonetti, A.; Intaglietta, I.; Sofo, A.; Gambacorta, E. Metal content of southern Italy honey of different botanical origins and its correlation with polyphenol content and antioxidant activity. Int. J. Food Sci. Technol. 2012, 47, 1909–1917. https://doi.org/10.1111/j.1365-2621.2012.03050.x.

[173] Khalil, M.; Sulaiman, S.A.; Alam, N.; Ramli, N.; Mohamed, M.; Bai'e, S.; Gan, S. Content and antioxidant properties of processed Tualang honey (AgroMas®) collected from different regions in Malaysia. Int. J. Pharm. Pharm. Sci. 2012, 4, 214–219.

[174] Sant'Ana, L.D.O.; Sousa, J.P.L.M.; Salgueiro, F.B.; Lorenzon, M.C.A.; Castro, R.N. Characterization of Monofloral Honeys with Multivariate Analysis of Their Chemical Profile and Antioxidant Activity. J. Food Sci. 2012, 77, C135–C140. https://doi.org/10.1111/j.1750-3841.2011.02490.x.

[175] Khalil, M.I.; Alam, N.; Moniruzzaman, M.; Sulaiman, S.A.; Gan, S.H. Phenolic Acid Composition and Antioxidant Properties of Malaysian Honeys. J. Food Sci. 2011, 76, C921–C928. https://doi.org/10.1111/j.1750-3841.2011.02282.x.

[176] Kishore, R.K.; Halim, A.S.; Syazana, M.S.N.; Sirajudeen, K.N.S. Tualang honey has higher phenolic content and greater radical scavenging activity compared with other honey sources. Nutr. Res. 2011, 31, 322–325. https://doi.org/10.1016/j.nutres.2011.03.001.

[177] Tezcan, F.; Kolayli, S.; Ulusoy, H.S.E.; Erim, F.B. Evaluation of organic acid, saccharide composition and antioxidant properties of some authentic Turkish honeys. J. Food Nutr. Res. 2011, 50, 33–40.

[178] Ulusoy, E.K.; Sarıkaya, S. Antioxidant and Antimicrobial Activity of Different Floral Origin Honeys from Türkiye. J. Food Biochem. 2010, 34, 321–335. https://doi.org/10.1111/j.1745-4514.2009.00332.x.

[179] Lachman, J.; Orsák, M.; Hejtmánková, A.; Kovářová, E. Evaluation of antioxidant activity and total phenolics of selected Czech honeys. LWT-Food Sci. Technol. 2010, 43, 52–58. https://doi.org/10.1016/j.lwt.2009.06.008.

[180] Mohamed, M.; Sirajudeen, K.N.S.; Swamy, M.; Yaacob, N.S.; Sulaiman, S.A. Studies on the Antioxidant Properties of Tualang Honey from Malaysia. Afr. J. Tradit. Complement. Altern. Med. 2009, 7, 59–63.

[181] Tuberoso, C.I.G.; Bifulco, E.; Jerković, I.; Caboni, P.; Cabras, P.; Floris, I. Methyl Syringate: A Chemical Marker of Asphodel (Asphodelus microcarpus Salzm. et Viv.) Monofloral Honey. J. Agric. Food Chem. 2009, 57, 3895–3900. https://doi.org/10.1021/jf803991j.

[182] Pichichero, E.; Canuti, L.; Canini, A. Characterisation of the phenolic and flavonoid fractions and antioxidant power of Italian honeys of different botanical origin. J. Sci. Food Agric. 2009, 89, 609–616. https://doi.org/10.1002/jsfa.3484.

[183] Krpan, M.; Marković, K.; Šarić, G.; Skoko, B.; Hruskar, M.; Vahcić, N. Antioxidant Activities and Total Phenolics of Acacia Honey. Czech. J. Food Sci. 2009, 27, S245–S247. https://doi.org/10.17221/1112-CJFS.

[184] Bertoncelj, J.; Doberšek, U.; Jamnik, M.; Golob, T. Evaluation of the phenolic content, antioxidant activity and colour of Slovenian honey. Food Chem. 2007, 105, 822–828. https://doi.org/10.1016/j.foodchem.2007.01.060.

[185] Blasa, M.; Candiracci, M.; Accorsi, A.; Piacentini, M.P.; Albertini, M.C.; Piatti, E. Raw Millefiori honey is packed full of antioxidants. Food Chem. 2006, 97, 217–222. https://doi.org/10.1016/j.foodchem.2005.03.039.

[186] Aljadi, A.M.; Kamaruddin, M.Y. Evaluation of the phenolic contents and antioxidant capacities of two Malaysian floral honeys. Food Chem. 2004, 85, 513–518. https://doi.org/10.1016/S0308-8146(02)00596-4.

[188] Bolanos de la Torre, A.A.S.; Henderson, T.; Nigam, P.S.; Owusu-Apenten, R.K. A universally calibrated microplate ferric reducing antioxidant power (FRAP) assay for foods and

applications to Manuka honey. Food Chem. 2015, 174, 119–123. https://doi.org/10.1016/j.foodchem.2014.11.009.

[197] Habib, H.M.; Al Meqbali, F.T.; Kamal, H.; Souka, U.D.; Ibrahim, W.H. Bioactive components, antioxidant and DNA damage inhibitory activities of honeys from arid regions. Food Chem. 2014, 153, 28–34. https://doi.org/10.1016/j.foodchem.2013.12.044.

[198] Ahmad, N.S.; Abdul Aziz, A.; Kong, K.W.; Hamid, M.S.A.; Cheong, J.P.G.; Hamzah, S.H. Dose–Response Effect of Tualang Honey on Postprandial Antioxidant Activity and Oxidative Stress in Female Athletes: A Pilot Study. J. Altern. Complement. Med. 2017, 23, 989–995. https://doi.org/10.1089/acm.2017.0129.
